# Bacteriophage tRNA-dependent lysogeny: requirement of phage-encoded tRNA genes for establishment of lysogeny

**DOI:** 10.1128/mbio.03260-23

**Published:** 2024-01-18

**Authors:** Carlos A. Guerrero-Bustamante, Graham F. Hatfull

**Affiliations:** 1 Department of Biological Sciences, University of Pittsburgh, Pittsburgh, Pennsylvania, USA; Weill Cornell Medicine, New York, New York, USA

**Keywords:** *Mycobacterium*, bacteriophage, lysogeny, tRNA

## Abstract

Bacteriophages are large and diverse components of the biosphere, and many phages are temperate. Upon infection, temperate phages can establish lysogeny in which a prophage is typically integrated into the bacterial chromosome. Here, we describe the phenomenon of tRNA-dependent lysogeny, a previously unrecognized behavior of some temperate phages. tRNA-dependent lysogeny is characterized by two unusual features. First, a phage-encoded tyrosine family integrase mediates site-specific recombination between a phage *attP* site and a bacterial *attB* site overlapping a host tRNA gene. However, *attP* and *attB* share only a short (~10 bp) common core such that a functional tRNA is not reconstructed upon integration. Second, the phage encodes a tRNA of the same isotype as the disrupted but essential host tRNA, complementing its loss, and consequently is required for the survival of lysogenic progeny. As expected, an integrase-defective phage mutant forms turbid plaques, and bacterial progeny are immune to superinfection, but they lack stability, and the prophage is rapidly lost. In contrast, a tRNA-defective phage mutant forms clear plaques and more closely resembles a repressor mutant, and lysogens are recovered only at very low frequency through the use of secondary attachment sites elsewhere in the host genome. Integration-proficient plasmids derived from these phages must also carry a cognate phage tRNA gene for efficient integration, and these may be useful tools for mycobacterial genetics. We show that tRNA-dependent lysogeny is used by phages within multiple different groups of related viruses and may be prevalent elsewhere in the broader phage community.

**IMPORTANCE:**

Bacteriophages are the most numerous biological entities in the biosphere, and a substantial proportion of phages are temperate, forming stable lysogens in which a prophage copy of the genome integrates into the bacterial chromosome. Many phages encode a variety of tRNA genes whose roles are poorly understood, although it has been proposed that they enhance translational efficiencies in lytic growth or that they counteract host defenses that degrade host tRNAs. Here, we show that phage-encoded tRNAs play key roles in the establishment of lysogeny of some temperate phages. They do so by compensating for the loss of tRNA function when phages integrate at an *attB* site overlapping a tRNA gene but fail to reconstruct the tRNA at the attachment junction. In this system of tRNA-dependent lysogeny, the phage-encoded tRNA is required for lysogeny, and deletion of the phage tRNA gives rise to a clear plaque phenotype and obligate lytic growth.

## INTRODUCTION

Bacteriophage lysogeny has been well characterized in bacteriophage lambda, and other temperate phages share several features, including a repressor that is required for lysogenic maintenance and shutting down lytic gene expression, and site-specific recombination systems for integration of the phage genome into the bacterial chromosome (1, 2). Several elaborations on these molecular schemes have been described, including integration-dependent immunity systems in which the phage attachment site is located within the repressor open reading frame (3, 4) and plasmid-like replication and partitioning systems that substitute for integration (5, 6). However, for most temperate phages, the mechanisms determining lysogenization frequency, prophage induction, and regulation of prophage integration and excision are not well understood.

Over 4,400 phages of bacterial hosts in the phylum Actinobacteria have been sequenced and annotated, providing a high resolution of viral diversity (7, 8). These can be readily sorted into groups of related genomes referred to as clusters (Clusters A, B, C, etc.), many of which can be divided into subclusters (Subclusters A1, A2, A3, etc.) based on sequence similarities (9); genomes with no close relatives are referred to as “singletons.” Approximately one-half of these phages were isolated on *Mycobacterium smegmatis,* and ~50% of these are temperate (8, 10). Although a subset of the temperate phages establishes prophages replicating extrachromosomally (5), most encode a site-specific recombinase of the tyrosine-integrase (Int-Y) or serine-integrase (Int-S) families (8). Examples of both systems have been investigated and used to construct integration-proficient plasmids for use in constructing stable single-copy *Mycobacterium* recombinants (11–13). A total of 13 distinct *attB* sites (designated attB1–attB-13) have been identified or predicted to be distributed around the *M. smegmatis* genome (14). Of these, 10 are used by Int-Y recombinases, and all of their *attB* sites overlap host tRNA or tmRNA genes (14). The phage and bacterial attachment sites (*attP* and *attB*, respectively) typically share a common sequence (the common core) of 30–45 bp that includes the 3′ half of the tRNA such that the tRNA is “reconstructed” at an attachment junction (*attL* or *attR*) following integration.

Bacteriophage genomes characteristically encode many protein-coding genes of unknown function, some of which are implicated in phage-bacterium interaction dynamics (15). Assortment of predicted actinobacteriophage-encoded proteins into “phamilies” and comparison of their genomic distributions illustrate the mosaic nature of phage genome architectures (8, 16). However, these phages also vary greatly in their tRNA gene content, with many carrying no tRNA genes and others with a near-complete coding set (17, 18). For example, the 104 Subcluster A2 phages are genomically closely related but vary in having between zero and five tRNA genes of various isotypes (19–21). The roles of these tRNA genes are unknown, although it has been proposed that they compensate for inadequacies of host tRNAs needed for lytic replication (22–27) or counteract tRNA-degradation-mediated phage defense systems (28, 29). However, it is unclear if these mechanisms alone explain the great diversity of tRNA gene repertoires seen among the actinobacteriophages.

Here, we describe a newly identified feature of temperate phage life cycles described as tRNA-dependent lysogeny. A subset of *Mycobacterium* temperate phages integrate at an *attB* site overlapping a host tRNA gene but do not reconstruct a functional tRNA at either *attL* or *attR*. Because mycobacteria have a minimal and non-redundant tRNA repertoire, integration would result in non-viable progeny, an outcome that is prevented by expression of a phage-encoded tRNA of the same isotype as the host tRNA gene at *attB*. Strikingly, deletion of the phage tRNA results in a clear plaque phenotype, a property observed for some naturally occurring phages lacking a tRNA gene such that prophage integration results in the non-viability of the host.

## RESULTS

### Identification of the *M. smegmatis attB* site for Subcluster K4 phages

**Fig 1:**
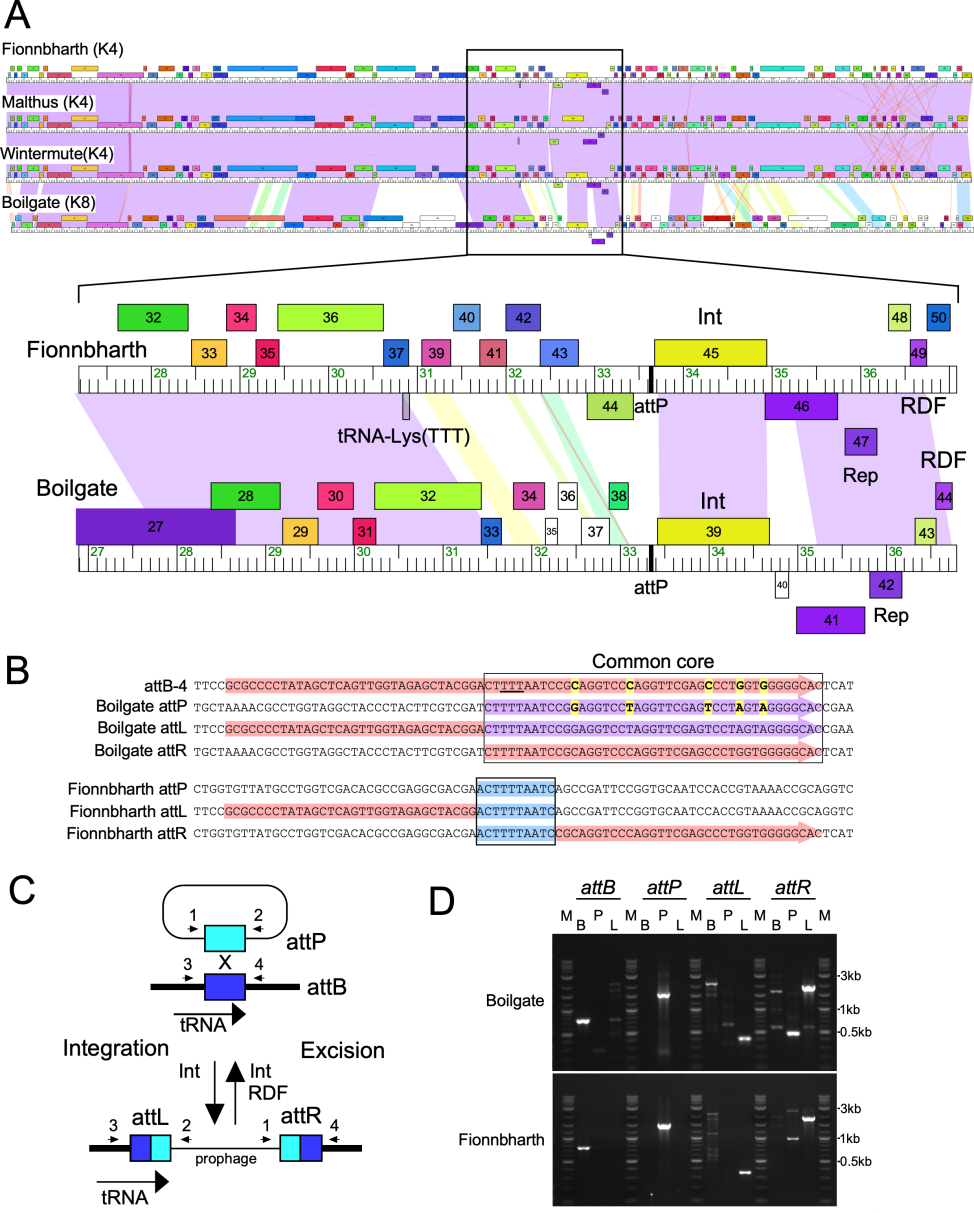
Cluster K4 phages use *M. smegmatis attB*-4 for site-specific integration. (**A**) Genome maps of Subcluster K4 mycobacteriophages Fionnbharth, Malthus, and Wintermute and Subcluster K8 mycobacteriophage Boilgate. The colored boxes correspond to protein-coding genes and are colored according to their phamily assignment (similarly colored are homologs). Shading between genomes reflects nucleotide sequence similarity and is spectrum colored with violet being the most similar and red the least similar above a threshold *E* value of 10^−4^. An expanded view of the central parts of the Fionnbharth and Boilgate genomes shows the integration functions, including the integrase gene (*int*) and attachment site, *attP*. (**B**) Alignments of the Boilgate and Fionnbharth attachment sites with *M. smegmatis* attB-4. The Boilgate *attP* site shares a 43-bp common core sequence (boxed) with attB-4 with five single-nucleotide differences (highlighted in yellow). attB-4 overlaps the 3′ end of a host tRNA-Lys-TTT gene (red highlighting; anticodon is underlined), and the Boilgate *attP* spans the 3′ half of the tRNA gene (purple highlighting). Strand exchange between the left end of the common core and the first mismatch in integrative recombination forms the *attL* and *attR* junction sites and a functional tRNA-Lys(TTT) at *attL*. Fionnbharth *attP* and its cognate *attL* and *attR* sites share a 10-bp common core (boxed; pale blue highlighted) with attB-4. (**C**) Schematic representation of phage integration. Int-mediated site-specific recombination occurs between an *attP* site in the phage genome (aqua box) and an *attB* site (blue box) in the bacterial genome; *attB* overlaps the 3′ half of a host tRNA gene. The products of the reaction are an integrated prophage flanked by attachment junctions *attL* and *attR*. The host tRNA is reconstituted at *attL* following integration. Primers used to PCR amplify attachment sites are numbered: 1 and 2, *attP*; 3 and 4, *attB*; 2 and 3, *attL*; 1 and 4, *attR*. Prophage excision is also mediated by Int but requires a recombination directionality factor (RDF). (**D**) PCR amplification of *attP, attB, attL,* and *attR* sites for Fionnbharth and Boilgate. For each PCR reaction, three templates were used, mc^2^155 (B), phage (P), and lysogen DNA (L), and primers amplified attachment sites as indicated. Expected PCR products for Boilgate: mc^2^155 *attB*, 770 bp; *attP*, 1,593 bp; *attL*, 415 bp; *attR*,1,949 bp. Fionnbharth: *attB*, 770 bp; *attP*, 1,503 bp; *attL*, 354 bp; *attR*, 1,921 bp. M, DNA marker.

Cluster K is a large group of mycobacteriophages (180 sequenced genomes) subdivided into eight subclusters (K1–K8) based on their sequence relationships (30). Cluster K phages are of general interest as they have broad host ranges, which include *M. tuberculosis* (31, 32). Previously, we reported that Subcluster K1 phages integrate into an *attB* site [attB-9, (14)] overlapping the *M. smegmatis* tmRNA gene (Msmeg_2093) (30). However, there is sequence variation among Cluster K integrase genes, and other Cluster K phages may use different *attB* sites (30, 33). Phages in Subclusters K3, K4, K6, K7, and K8 encode closely related Int-Ys (within the same phamily) but are distinct from Subclusters K1 and K2 Int-Ys (~25% aa identity). A bioinformatic search of the Subcluster K8 phage Boilgate—which is syntenic with other Cluster K phages (Fig. 1A)—identified a 43-bp region with similarity to the *M. smegmatis* genome, although with five single-nucleotide differences (Fig. 1B). This region is situated immediately upstream of Boilgate *int* (Fig. 1A), and the *M. smegmatis* region overlaps the 3′ half of a tRNA-Lys-TTT gene (Msmeg_5758, Fig. 1B and C); none of the five single-nucleotide differences prevent folding of a predicted functional tRNA at *attL*. These regions thus likely correspond to Boilgate *attP* and *attB*, respectively, with the chromosomal site designated previously as attB-4 (Fig. 1B) (14); inspection of Boilgate *attP* identified putative arm-type Int-binding sites flanking the common core (Fig. S2), and Boilgate integration at attB-4 was confirmed by PCR (Fig. 1C and D). In a similar bioinformatic search for *att* sites used by Subcluster K4 phages, such as Fionnbharth, we failed to identify similar regions of identity with the *M. smegmatis* genome. Presumably, the common core is either atypically small or highly divergent. The Boilgate and Fionnbharth integrases are sufficiently similar (78% aa identity) to suggest they may use the same *attB* site.

To identify the Fionnbharth integration sites, we PCR amplified DNA from a Fionnbharth lysogen using a phage-specific primer and a random primer. PCR amplification and sequencing of *attL* and *attR* confirmed that Fionnbharth integrates into attB-4 (Fig. 1C and D). Alignment of the phage sequences shows that Fionnbharth *attP* and *attB* have a small common core (10 bp; Fig. 1B), with putative arm-type Int-binding sites flanking the Fionnbharth *attP* core (Fig. S1); *attL* and *attR* sequencing confirmed that strand exchange occurs within the common core. This observation was surprising because *M. smegmatis* has a minimal tRNA gene repertoire with only a single tRNA gene with a tRNA-Lys-TTT isotype. Disruption of the tRNA gene by phage integration thus is expected to yield non-viable progeny.

### Fionnbharth-encoded tRNA-Lys-TTT complements integrative disruption of the host tRNA

**Fig 2:**
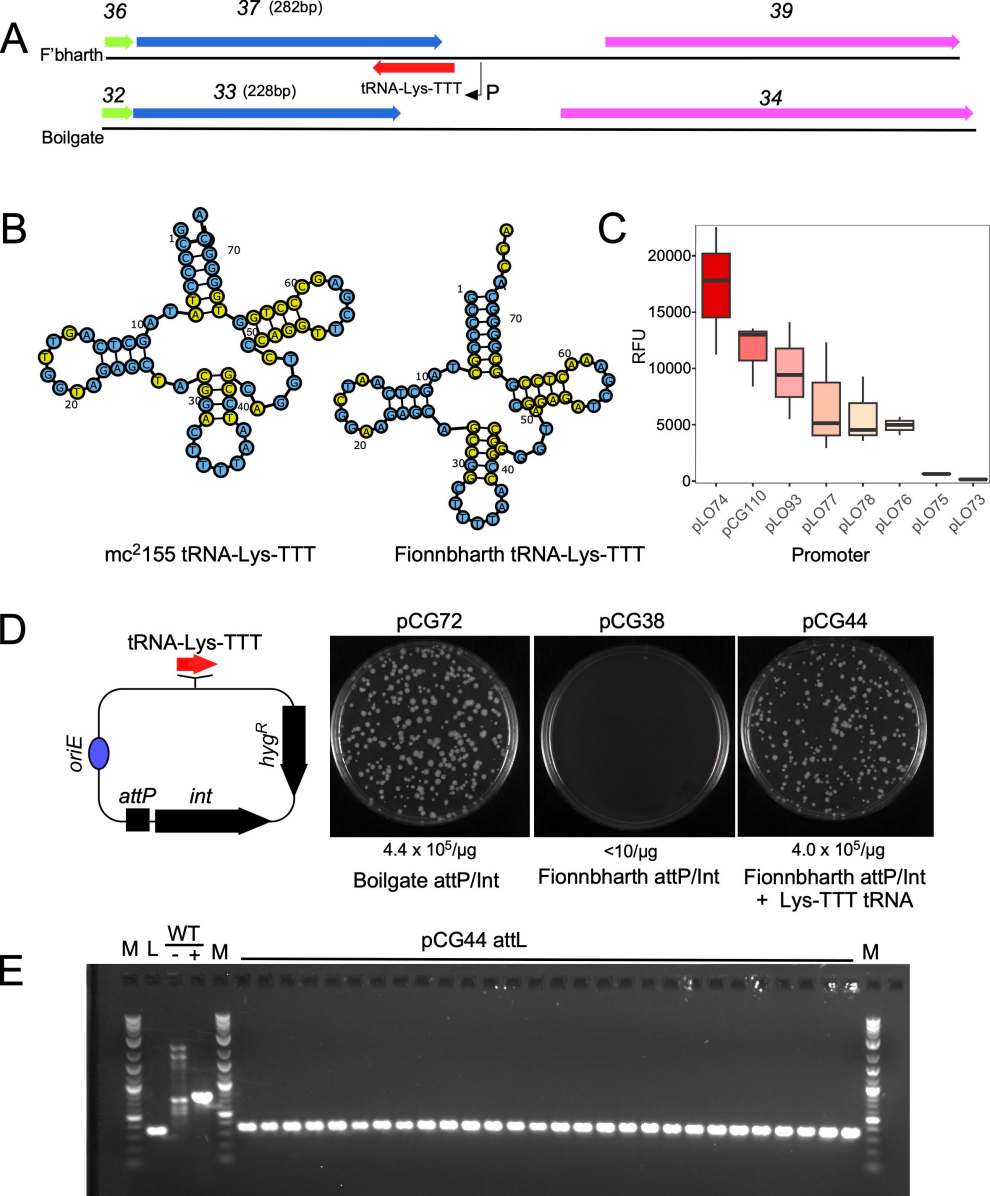
A Fionnbharth-encoded tRNA-Lys-TTT is required for viable integration. (**A**) Organization of the Fionnbharth and Boilgate genomes containing the tRNA-Lys-TTT gene. Fionnbharth encodes a tRNA-Lys-TTT gene that overlaps gene *37* and is transcribed from the opposite strand. Boilgate lacks the tRNA gene. (**B**) Comparison of the predicted secondary structures of the *M. smegmatis* mc^2^155 and Fionnbharth tRNA-Lys-TTT genes. Bases differing between the two tRNAs are shown in yellow. (**C**) Box and whisker plots showing relative fluorescence units (RFU) of mCherry activity from reporter plasmids in *M. smegmatis*. Plasmid pCG110 carries a 136-bp fragment from upstream of the Fionnbharth tRNA gene and is ~75% as active as the hsp60 promoter in plasmid pLO74 and stronger than a calibrated set of synthetic mycobacterial promoters; pLO73 is the promoter-less vector (34). Each plot is from three biological replicates; boxes indicate interquartile range; lines inside boxes show median values, whiskers indicate minimum and maximum values 1.5 times the value of the interquartile range. (**D**) Left, genetic structure of integration-proficient plasmids is shown, either with (e.g., pCG44) or without (e.g., pCG38) a phage-derived tRNA-Lys-TTT gene (red arrow). Right, transformation of integrative plasmids in *M. smegmatis*. Plasmids used for transformation (pCG72, pCG38, and pCG44) are indicated, and the frequencies of transformation are shown below. The pCG72 and pCG44 plates reflect recovery after electroporation of approximately 10 ng DNA with average efficiencies of 4 × 10^5^ transformants/µg DNA, whereas for pCG38, the entire transformation mix—equivalent to electroporation of 100 ng DNA—was plated. (E) PCR amplification of *attL* in 28 independent pCG44 transformants demonstrating transformation mediated *via attP* × *attB* site-specific recombination. M, 1-kb plus DNA ladder; L, Fionnbharth lysogen with attL primers; WT, negative (−) and positive (+) controls amplified with attL primer pair and attB primer pairs, respectively. Fionnbharth lysogen control gives predicted attL product at 354 bp, while *M. smegmatis* mc^2^155 yields unspecific PCR products (negative control) with attL primers and expected product at 770 bp with attB primer pair. All independent pCG44 transformants have the expected attL product at 354 bp.

Examination of the Fionnbharth genome (and all other Subcluster K4 phages) shows that it encodes its own tRNA-Lys-TTT gene. This suggests that the phage-encoded tRNA may complement loss of the host tRNA-Lys-TTT upon integration. Curiously, the Fionnbharth tRNA-Lys-TTT overlaps protein-coding gene *37* but is transcribed from the opposite DNA strand (Fig. 2A). The Fionnbharth and Boilgate genomes are generally similar in this region but with divergence at the 3′ end of Fionnbharth *37* and Boilgate *33*. This corresponds to the Fionnbharth tRNA location and results in Fionnbharth gp37 being C-terminally extended by 18 amino acids relative to Boilgate gp33 (Fig. 2A). The functions of Fionnbharth gene *37* and its Boilgate homolog are not known, but related genes are present in other Clusters E, K, L, and Y and singleton mycobacteriophages. However, only Subcluster K4 phages have a tRNA overlapping the 3′ end of the gene, and all 17 Subcluster K4 phages have no more than 17 bp identity with attB-4, none spanning the complete 3′ end. The origins of the Fionnbharth tRNA are unclear. It may have been acquired from another phage or from a bacterial genome, although it is not closely related to the *M. smegmatis* tRNA-Lys-TTT (Fig. 2B). The closest relatives are in *Mycobacterium moriokaense* and *Mycobacterium rutilum* (88% identical to both), and it is not known if Subcluster K4 phages infect these strains. The presence of a promoter for expression of the Fionnbharth tRNA was demonstrated by inserting the upstream region into a reporter plasmid and showing strong expression relative to a calibrated set of artificial promoters (34).

To test whether the phage-encoded tRNA is required for viability following integration, we constructed Fionnbharth-derived integration-proficient plasmids either with (pCG44) or without (pCG38) the Fionnbharth tRNA-Lys-TTT gene and its presumed upstream expression signals (Fig. 2D). The integration-proficient plasmids consist of the integrase gene, the upstream intergenic region containing *attP*, a hygromycin resistance cassette, and an *E. coli* origin of replication that is not functional in mycobacteria (Fig. 2D); a Boilgate-derived plasmid (pCG72) carrying its *attP-int* region was also constructed. Plasmid pCG72 (Boilgate *attP-int*) efficiently transforms *M. smegmatis*, whereas no transformants were recovered with pCG38 (Fionnbharth *attP-int*). However, pCG44, which additionally carries the Fionnbharth tRNA-Lys-TTT, transforms as efficiently as pCG72 (Fig. 2D). To confirm that pCG44 transforms via integrative site-specific recombination, we PCR amplified the predicted *attL* junction from 28 independent transformants. All of the transformants gave a product indicating recombination between *attP* and attB-4 (Fig. 2E). These observations suggest that Fionnbharth integration destroys functionality of the host tRNA-Lys-TTT gene, and the defect is complemented by expression of a phage tRNA with the same isotype.

### The Fionnbharth tRNA-Lys-TTT is required for efficient lysogeny

**Fig 3:**
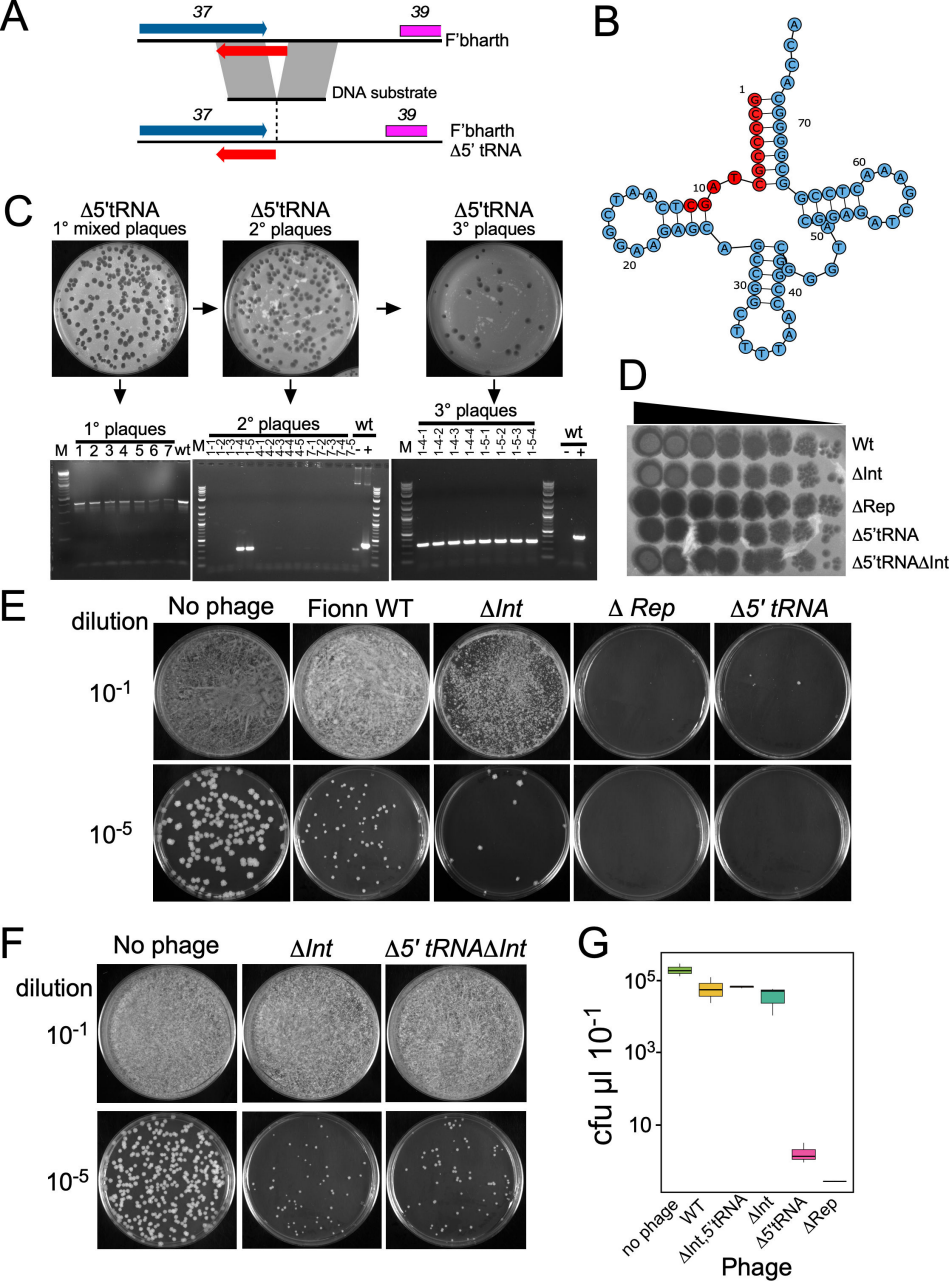
Fionnbharth-encoded tRNA-Lys-TTT is required for efficient establishment of lysogeny. (**A**) Construction of a mutant Fionnbharth derivative with deleted tRNA. A DNA substrate was designed that includes the 3′ end of gene *37* and sequences upstream of the tRNA gene but lacks the 11 bp 5′ end of the tRNA; hence, the 11 bp following the stop codon of gene 37 is deleted. The DNA substrate is 200 bp with the deletion centrally positioned. (**B**) Secondary structure of the tRNA-Lys-TTT with the deleted region shown in red. (**C**) Co-electroporation into recombineering-proficient cells of Fionnbharth genomic DNA and the DNA substrate shown in panel A yielded primary plaques that were screened using PCR with primers flanking the deleted region. However, the wild-type and mutant PCR products are too similar in size to resolve. Three randomly chosen primary plaques were re-plated, and five secondary plaques of each were screened using Mismatch Amplification Mutation Assay-PCR (MAMA-PCR) in which only the mutant gives a product; plaques 1–4 and 1–5 are positive. PCR with wild-type Fionnbharth DNA (WT) shows only weak amplification with MAMA-PCR primers (–) but strong amplification with matching primers (+). Secondary plaques 1–4 and 1–5 were re-plated, and four tertiary plaques of each tested positive with MAMA-PCR primers. (**D**) Spot dilution series of Fionnbharth and mutant derivatives on a lawn of *M. smegmatis*. Wild-type and Δ*Int* phages form evidently turbid plaques, whereas Δ*int* and Δ5′tRNA mutants form clear plaques. The Δ*int*Δ5′tRNA double mutant forms plaques more turbid than either the Δ*int* or Δ5′tRNA mutants. The agar plate was incubated for 5 days at 37°C. (**E and F**) Establishment of lysogeny assessed by plating 10-fold or 10^−5^-fold (as indicated) dilutions of bacterial cultures on solid media seeded with 2 × 10^9^ phage particles as indicated. Representative images of three independent transformation experiments are shown. (G) Box and whisker plots of lysogenization experiments (*N* = 3) with Fionnbharth and mutant derivatives. Colony-forming units (CFU) per microliter of bacterial culture surviving phage infection are shown; boxes indicate interquartile range, lines inside boxes indicate median values, whiskers indicate minimum and maximum values 1.5 times the value of the interquartile range.

The behavior of the integration-proficient plasmids suggests that the phage tRNA-Lys-TTT gene is required for normal establishment of lysogeny and, perhaps, is not required for lytic growth. To test this, we constructed a Fionnbharth derivative (Δ5′tRNA) in which the 11 bp at the extreme 5′ end of the tRNA gene is deleted, leaving the overlapping gene *37* intact (Fig. 3A and B). Using Bacteriophage Recombineering on Electrporation DNA (BRED) recombineering (35), primary plaques were obtained containing mutant derivatives, plaque-purified mutant secondary derivatives were identified by PCR, and a homogenous lysate made after a third round of purification (Fig. 3C). Mutant and wild-type plaques are similarly sized, and we conclude that the tRNA-Lys-TTT is not required for lytic growth (Fig. 3D). We also constructed Fionnbharth derivatives in which either the integrase (Δ*int*) or repressor (Δ*rep*) genes are deleted, which grow well lytically as expected (Fig. 3D; Fig. S2).

The plaque morphologies of these mutant phages are informative (Fig. 3D). Wild-type Fionnbharth forms turbid plaques (and spots) typical of temperate phages, and the Δ*rep* mutant forms clear plaques as expected (Fig. 3D). The Δ*int* mutant forms turbid plaques similar to wild-type Fionnbharth, indicating prophage integration is not required for superinfection immunity, even though it is presumably needed for stable lysogeny (Fig. 3D). Interestingly, the Δ5′tRNA mutant forms plaques almost as clear as the Δ*rep* mutant, suggesting that the tRNA-Lys-TTT gene is required for lysogeny and that integration of the Δ5′tRNA mutant fails to yield viable progeny. To explore this further, we constructed a double mutant in which both the 5′tRNA region and the integrase gene are deleted (Fig. S2). The double mutant phenotypically resembles the Δ*int* mutant and forms plaques considerably more turbid than either the Δ*rep* or Δ5′tRNA mutants (Fig. 3D).

**Fig 4:**
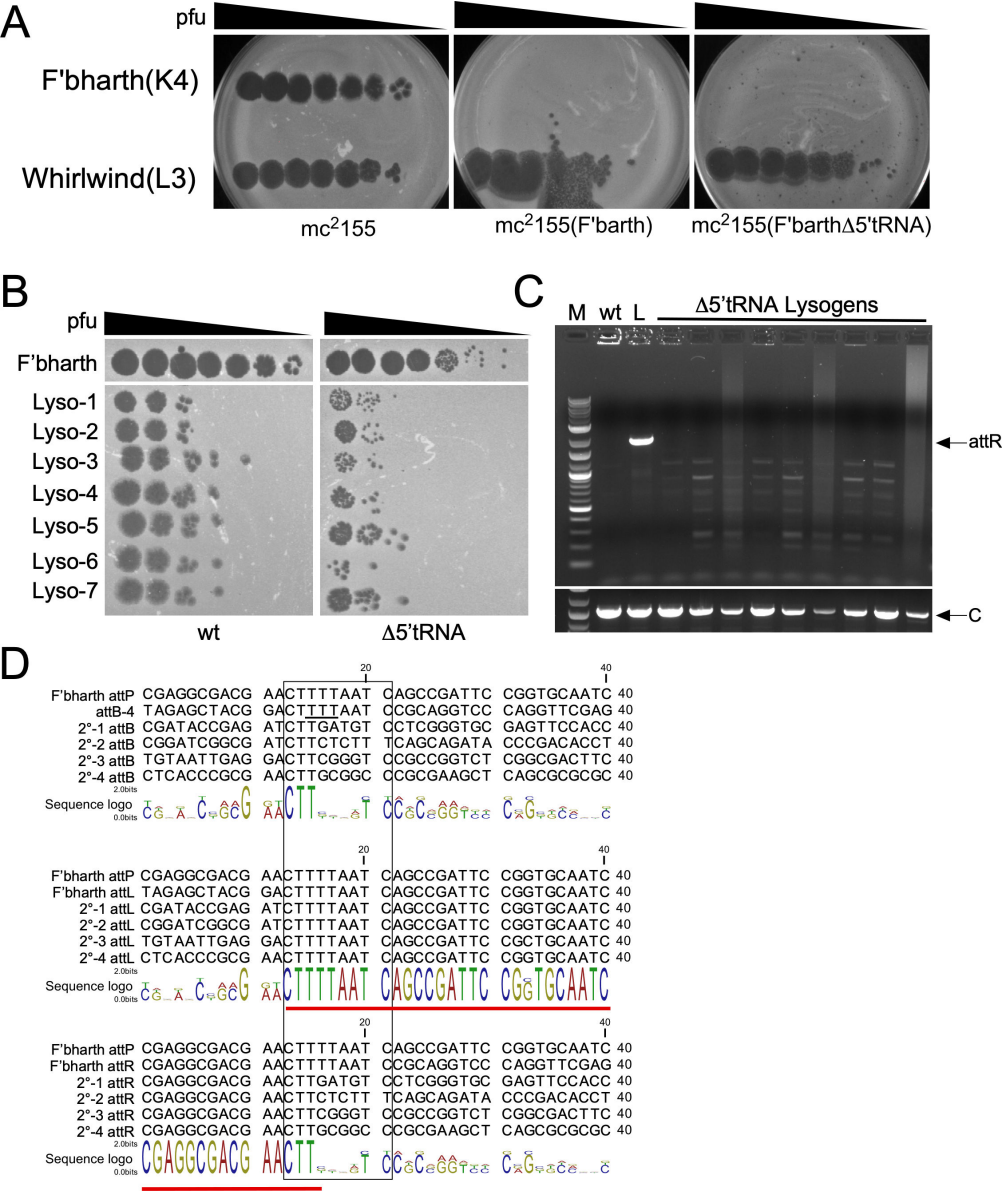
Fionnbharth Δ5′tRNA integrates at secondary *attB* sites in the *M. smegmatis* genome. (**A**) Serial 10-fold dilutions (10^−1^ to 10^−7^) of Fionnbharth and Whirlwind phage lysates were spotted on lawns of WT *M. smegmatis* mc^2^155, a *M. smegmatis* Fionnbharth lysogen, and a putative *M. smegmatis* Fionnbharth Δ5′tRNA lysogen. Both lysogens are immune to Fionnbharth superinfection but not to the unrelated phage Whirlwind (Subcluster L3). (**B**) Supernatants from cultures of seven putative wild-type lysogens and seven Δ5′tRNA putative lysogens were filtered, 10-fold serially diluted, and spotted onto lawns of *M. smegmatis* mc^2^155; Fionnbharth was similarly diluted and plated (top row). (**C**) *attR* PCR analysis of 5′tRNA lysogens demonstrating that they do not have prophages integrated into attB-4. M, NEB 1-kb plus marker; WT and L, *M. smegmatis* mc^2^155 and Fionnbharth lysogen with *attR* primers, respectively; arrow indicates *attR*. Δ5′tRNA lysogens are amplified with *attR* primers. Bottom panel shows control (**C**) PCR of a common chromosomal locus. (**D**) Alignments of Fionnbharth *attB* and attP with secondary *attB, attL,* and *attR* sites of four independent Δ5′tRNA lysogens (2°−1, 2°−2, 2°−3, 2°−4). The sequence logo below each alignment shows the consensus sequence and conservation at each position. Stacked letters indicate positions where more than one base is utilized. The *attP*-derived sequences within *attL* and *attR* are underlined in red. The anticodon of the tRNA-Lys-TTT at attB-4 is underlined in black.

To assess lysogenization frequencies under a standard set of conditions, a culture of *M. smegmatis* was diluted and plated onto solid media seeded with wild-type Fionnbharth or the various mutant derivatives we constructed. When plated on wild-type Fionnbharth, the number of surviving colonies ranged from 20% to 80% of that without phage selection (Fig. 3E), and all seven colonies we tested are lysogenic by superinfection immunity and phage release (Fig. 3; see also Fig. 4). In contrast, few colonies were recovered when the culture was plated onto media seeded with the Δ*rep* mutant (Fig. 3E). The frequency of colony recovery was reduced only ~10-fold in the Δ*int* mutant, and as expected, when replated and retested (Fig. 3E), the survivors do not confer immunity and are not stably lysogenic (Fig. S3); this is consistent with a model in which integrase is required for stable lysogeny, but not for superinfection immunity, as seen in phage lambda (36). As predicted from the plaque morphotypes, colony survival was greatly reduced for the Δ5′tRNA mutant and more closely resembles a Δ*rep* mutant than a Δ*int* mutant (Fig. 3E). This is consistent with the interpretation that the phage tRNA-Lys-TTT is required for viability of Fionnbharth lysogens (Fig. 3E). As anticipated, cell survival increases dramatically if *int* is also deleted, and the number of survivors is similar to that seen with the Δ*Int* mutant, as further evidence that integration is responsible for the lethal phenotype observed with the Δ5′tRNA mutant (Fig. 3F). A summary of lysogenization frequencies is shown in Fig. 3G, and we conclude that Fionnbharth clearly requires the tRNA-lys-TTT gene for efficient lysogenization. We will refer to this phenomenon as tRNA-dependent lysogeny.

### Phage-encoded tRNA inactivation results in integration at secondary *attB* sites

The recovery of *M. smegmatis* survivors following plating on the Δ5′tRNA mutant—albeit at low frequency—could be accounted for by integration events at secondary *attB* sites giving rise to viable progeny. Seven independent putative Δ5′tRNA lysogens were colony purified, and all are immune to superinfection (Fig. 4A; Fig. S2) and release phage particles into culture supernatants, although on average the titers of spontaneously released phage are about 20-fold lower than wild-type lysogens (Fig. 4B). PCR analyses confirmed that the Δ5′tRNA prophage is not integrated at attB-4 (Fig. 4C).

To determine the sites of Δ5′tRNA integration, we used a similar strategy as for wild-type Fionnbharth, using PCR with a Fionnbharth-specific primer and a random primer. Four independent lysogens were characterized, and the PCR products were sequenced to identify the integrative junctions. The sites were further characterized using PCR primers to specifically amplify *attL* and *attR*, sequencing the PCR products, and deducing the *attB* sites being used. We determined four secondary *attB* sites with this approach (2°−1, 2°−2, 2°−3, 2°−4), all of which lie within protein-coding genes that are predicted to be non-essential for viability (37) (Table 1; Fig. 4D). Alignment of the secondary *attB* sites with attB-4 shows very few well-conserved nucleotide positions, except for the 5′ part of the common core (5′-CTT) present in all sites (Fig. 4D). Notably, not all of the 10-bp Fionnbharth common core is present in all secondary sites, and the 6–8 bp that typically constitute the overlap region between the scissile bonds for integrase-mediated DNA cleavage (38) are not conserved. Base mismatches between the recombination partners are typically strongly deleterious to recombination (39), and either that is not true for Fionnbharth integrase-mediated recombination, or there is a shorter overlap region with the scissile bonds spaced only 3 bp apart. Analysis of the *attL* and *attR* sites resulting from secondary site integration shows no indication of mismatch tolerance, and it seems plausible that Fionnbharth integrase atypically cleaves its DNA sites with just a 3-bp (5′-CTT) overlap (Fig. 4D). We also note that few bases are conserved among secondary *attB* sites outside of the common core that might indicate nucleotides important for integrase recognition. However, interpretation of the sequences relevant for recombination should be cautious, given that these events arise at a frequency of <10^−5^ of wild-type Fionnbharth lysogenization. We note that some tyrosine integrases tolerate mismatches in the overlap region, most notably Bacteroides CtnDOT and NBU1 (40, 41). In NBU1, the *attP*/*attB* core overlaps the 3′ end of a leucine tRNA (41) and not the anticodon loop as is common for *attP*/*attB* sites in mycobacteriophages.

**TABLE 1:** Secondary *attB* sites used by Fionnbharth Δ5′tRNA

2° attB	Coordinate^*a*^	Orientation^*b*^	Gene^*c*^	Function
1	6278819	+	MSMEG_6215	Hypothetical protein
2	2870515	−	MSMEG_2807	Response regulator transcription factor
3	3181782	+	MSMEG_3106	Quinone oxidoreductase
4	5343802	+	MSMEG_5250	Hypothetical protein

^*a*^ The coordinate given is the leftmost position of the common core.

^*b*^ The orientation given is the orientation of the common core within the genome plus (+) or minus (−) strand.

^*c*^ Each secondary *attB* site lies within the coding region of the genes shown.

### Cluster L phages also integrate at attB-4 and use tRNA-dependent lysogeny

**Fig 5:**
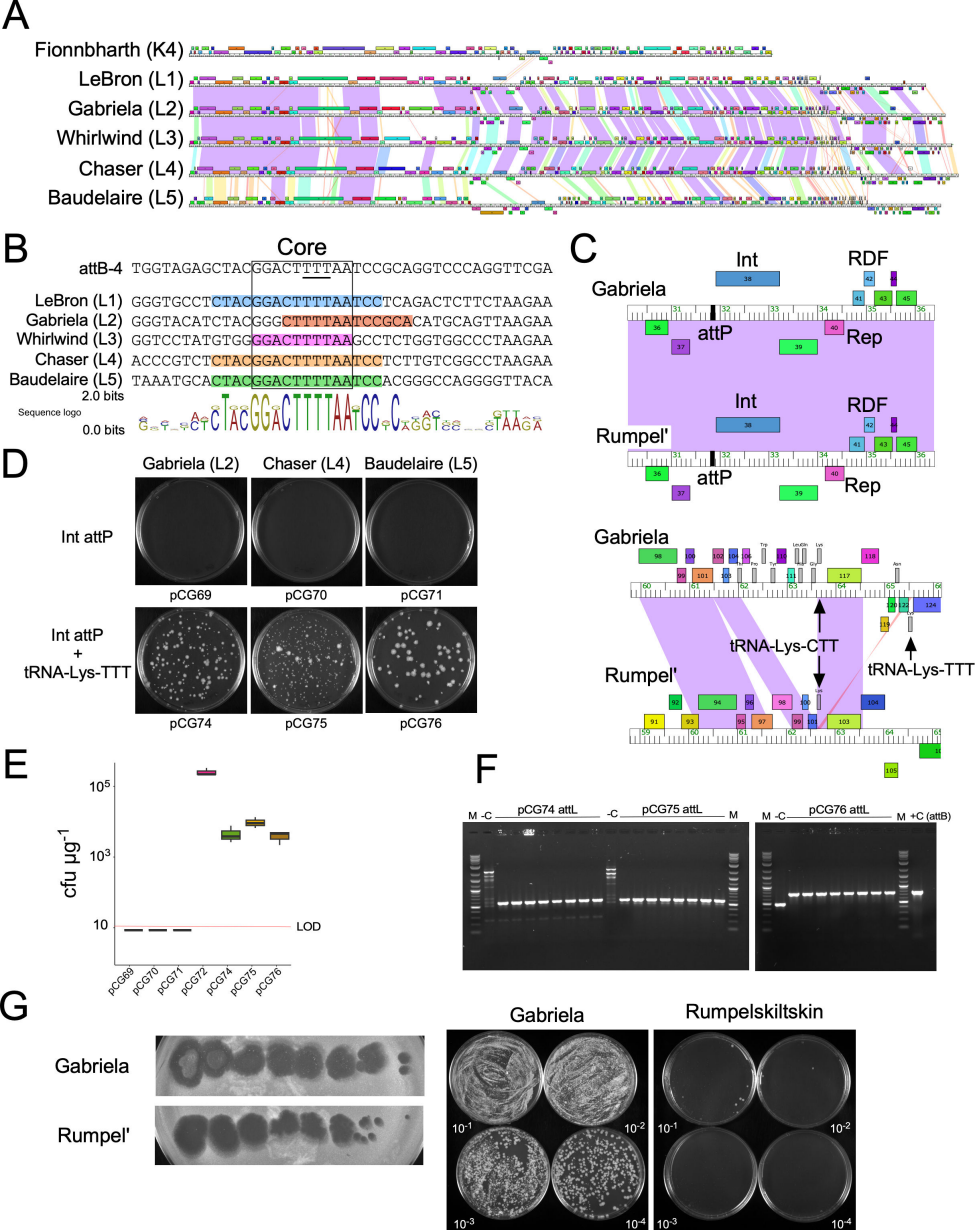
tRNA-dependent lysogeny by cluster L mycobacteriophages. (**A**) Genome maps of Fionnbharth (Subcluster K4) and Cluster L phages Lebron, Gabriela, Whirlwind, Chaser, and Baudelaire; subcluster assignments are shown in parentheses. Genomes are represented as described for Fig. 1A. (**B**) Alignment of the core sequences of *M. smegmatis* mc^2^155 attB-4 and the *attP* sites of Subcluster L1–L5 representative phages. The highlighted sequences indicate positions that are identical between *attP* sites and attB-4; the anticodon of the tRNA-Lys-TTT at attB-4 is underlined. The sequence logo below each alignment indicates the consensus sequence and conservation of each position. Stacked letters indicate positions where more than one base is utilized. The minimal common core shared by all sequences is boxed. (**C**) Segments of Gabriela and Rumpelstiltskin genomes (both in Subcluster L2) showing the integration and immunity system (top) and the tRNA regions (bottom). The Gabriela and Rumpelstiltskin integration and immunity regions are 98% as indicated by violet-colored nucleotide sequence similarity between the genomes. The two genomes vary in the tRNA regions, with deletions in Rumpelstiltskin removing all of its tRNA genes except for a tRNA-Lys-CTT gene. (**D**) Transformation of *M. smegmatis* with integration-proficient plasmids (as in Fig. 2C) derived from phages Gabriela (L2), Chaser(L4), and Baudelaire (L5). Plasmids pCG74, pCG75, and pCG76 all carry a phage-derived tRNA-Lys-TTT gene, whereas plasmids pCG69, pCG70, and pCG71 do not. (**E**) Box and whisker plots for transformation efficiencies of integration-proficient plasmids shown in Fig. 5D. Plasmids pCG69, pGD70, and pCG71 transform below the limit of detection of 10 (LOD, red line). (**F**) PCR analysis of *attL* junctions in pCG74, pCG75, and pCG76 transformants using four small and four larger colonies of each pCG74 and pCG75 and eight uniform colonies for pCG76. M denotes the 1-Kb plus ladder (NEB); −C indicates where *M. smegmatis* mc^2^155 has been used as template with attL primers of Gabriela, Chaser, and Baudelaire (Table S3). Non-specific products are observed for “−C” reactions. “+C” represents where *attB* primers are used to amplify *M. smegmatis* (770 bp). Products of *attL* amplification are observed at 590 bp for pCG74, 588 bp for pCG75, and 765 bp for pCG76 for all transformants as expected for each *attL* junctions of Gabriela, Chaser, and Baudelaire, respectively. (**G**) Serial dilutions of phages Gabriela and Rumpelstiltskin were plated on a lawn of *M. smegmatis* to show plaque morphotypes (left), and lysogenization of *M. smegmatis* by Gabriela but not Rumpelstiltskin is illustrated at the right, performed as in Fig. 3E.

In search of other mycobacteriophages using tRNA-dependent lysogeny, we identified Cluster L phages as good candidates, lacking extended (>35 bp) nucleotide identity between phage and bacterial genomes, and encoding several tRNA genes. Although Cluster L phages are generally not closely related to Cluster K phages (Fig. 5A), they code for similar integrases, and in one example, LeBron (Subcluster L1) Int has 48% amino acid sequence identity to Fionnbharth Int. A BLASTN search of the region upstream of LeBron *int* identified a 17-bp segment identical to the *M. smegmatis* genome, which lies wholly within the tRNA-Lys-TTT gene (Fig. 5B); putative arm-type Int-binding sites flanking this putative core were also found (Fig. S4). There is substantial variation among the different subclusters within Cluster L in these parts of their genomes; however, they are closely related within each subcluster, and we chose representatives to illustrate the segments of nucleotide similarity to attB-4 (Fig. 5B). Notably, Whirlwind (Subcluster L3) shares only 10 bp of identity with attB-4, 8 bp of which are also shared with Fionnbharth *attP*. PCR amplification showed that prophages of Baudelaire are integrated into the attB-4 site of *M. chelonae* (Sally Molloy, personal communication).

Unlike the Subcluster K4 phages, Cluster L phages often carry several tRNA genes, with nine in LeBron (Subcluster L1) and 17 in Baudelaire (Subcluster L5) (Fig. S5; Table S1); some, such as Rumpelstiltskin (Subcluster L2), have deletions in which most of the tRNA genes are lost (Fig. 5C; Fig. S5). Most of the 72 Cluster L phages code for a tRNA-Lys-TTT, although phages Rumpelstiltskin and Bromden (Subclusters L2 and L4, respectively) do not. Typically, most of the Cluster L phage tRNA genes are clustered and rightward transcribed, with the exception being the tRNA-Lys-TTT gene which is leftward transcribed (Fig. 5C; Fig. S5). The rightward-transcribed clustered tRNA genes are likely expressed in lytic growth, but we predict the leftward-transcribed genes—including the tRNA-Lys-TTT—are lysogenically expressed. A notable exception is Baudelaire where the tRNA-Lys-TTT is clustered among the other tRNA genes (Fig. S5).

To test whether viable integration of Cluster L phages is dependent on the phage-encoded tRNA-Lys-TTT, we constructed integration-proficient plasmids either with or without their cognate phage-encoded tRNA-Lys-TTT genes (Fig. 5D). Plasmids derived from Gabriela, Chaser, and Baudelaire all fail to transform *M. smegmatis* unless they also carry a phage tRNA-Lys-TTT gene (Fig. 5D), although the transformation frequencies are somewhat lower than for the Boilgate-derived plasmid, pCG72 (Fig. 5E). Although the colony morphology and size of the transformants are variable, we confirmed by PCR (Fig. 5F) that the plasmids integrated by site-specific recombination between *attP* and *attB*. We speculate that the difference in colony morphology and size could result from tandem integration events in which a second plasmid integrates using the plasmid tRNA gene as an *attB* site.

Curiously, Rumpelstiltskin and Bromden lack a tRNA-Lys-TTT gene, and we surmised these phages either use an alternative *attB* site for integration, have a different mechanism for accommodating disruption of the host tRNA-Lys-TTT, or they do not lysogenize. In contrast to Gabriela, Rumpelstiltskin forms clear plaques, and we observed no lysogenic survivors following plating of *M. smegmatis* on a phage-seeded plate (Fig. 5G). Rumpelstiltskin behaves similarly to the Fionnbharth 5′tRNA mutant, consistent with the requirement of the phage tRNA-Lys-TTT for lysogeny. We note that the only tRNA gene in Rumpelstiltskin is a tRNA-Lys-CTT gene (Fig. 5C), which does not complement the host tRNA disruption.

### Other actinobacteriophages using tRNA-dependent lysogeny

**Fig 6:**
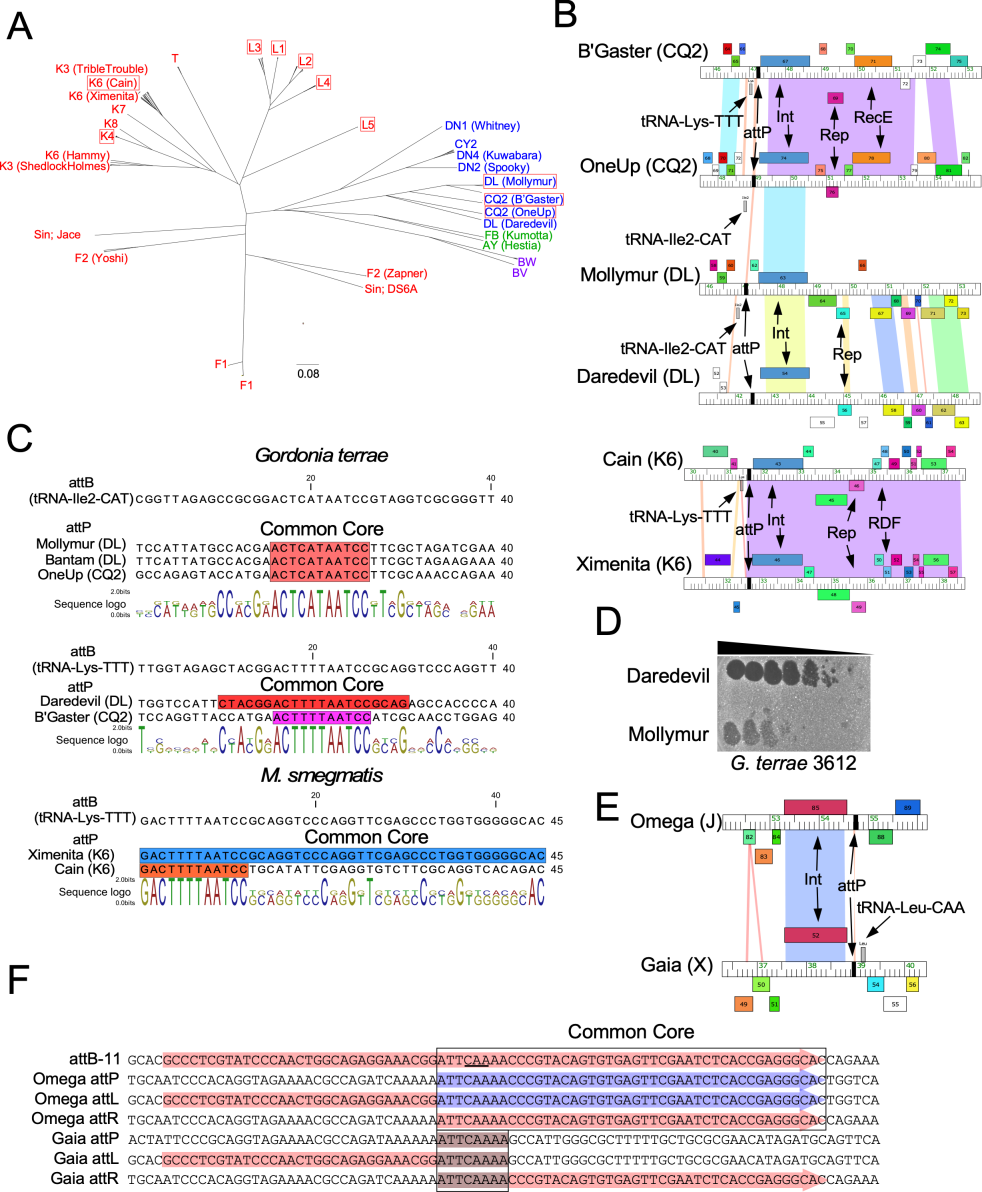
Other actinobacteriophages using tRNA-dependent lysogeny. (**A**) Maximum likelihood phylogenetic tree of a phamily of integrase proteins. Cluster/subcluster phage designations are shown at the nodes and are colored according to the bacterial host genus: red, *Mycobacterium*; blue, *Gordonia*; green, *Arthrobacter*; purple, *Propionibacterium*. Phages using tRNA-dependent lysogeny are shown in red boxes. Note that phages within some clusters/subclusters can code for integrases in more than one phamily, and in those instances, an example phage is indicated in parentheses. Jace and DS6A are singleton genomes (Sin). Scale bar indicates amino acid substitutions/site distance. (**B**) Phage integration/immunity regions of several phages that use tRNA-dependent lysogeny and their relatives. Genome maps are shown as described for Fig. 1A. *Gordonia* phages, BrutonGaster/Oneup (CQ2) and Mollymur (DL), use tRNA-dependent lysogeny; Daredevil (DL) does not reconstruct the host tRNA at *attL* and also lacks a phage-encoded tRNA-Ile2-CAT. Subcluster K6 phage Cain uses tRNA-dependent lysogeny, but Ximenita (K6) reconstructs the host tRNA at *attL* and does not encode its own tRNA. (**C**) Alignments of *attB* and *attP* for *Gordonia* phages in Clusters CQ2 and DL that use tRNA-dependent lysogeny, and *Mycobacterium* phages Cain and Ximenita in Subcluster K6; Cain uses tRNA-dependent lysogeny, but Ximenita reconstructs the host tRNA at *attL*. Colored boxes indicate sequence identity (Core) between *attB* and attP. The sequence logo below each alignment shows the consensus sequence and the frequency at which a base is used at each position. (**D**) Tenfold serial dilutions of Daredevil and Mollymur lysates plated on *G. terrae*. (**E**) Portion of genome maps showing integrase and attP for Omega (Cluster J) and Gaia (Cluster X). The phages are not closely related, but their integrases share 68% nucleotide identity (blue shading). The Omega and Gaia attP sites are inverted relative to rest of the genomes. Cluster J phages integrate at attB-11 overlapping tRNA-Leu-CAA. Gaia encodes a tRNA-Leu-CAA tRNA downstream of Int, but Omega does not. (F) Alignment of the Omega (J) and Gaia (X) attachment sites. The Omega *attP* site shares a 44-bp common core sequence (boxed) with attB-11; attB-4 overlaps the 3′ end of the host tRNA-Leu-CAA gene (red highlighting; anticodon is underlined), and the Omega *attP* spans the 3′ half of the tRNA gene (purple highlighting). Integration gives a functional tRNA at *attL*. Gaia *attP* shares only an 8-bp core with attB-11 (brown highlighting) and fails to form a functional tRNA at *attL*.

To look for additional examples of tRNA-dependent lysogeny among actinobacteriophages, we investigated the collection of 4,400 sequenced phages for those encoding a tyrosine integrase, have sufficiently small common cores that the *attB* site cannot be confidently predicted bioinformatically, and also code for one or more tRNA genes. First, we explored the ~280 phages with integrases related to those in Fionnbharth and LeBron, which are found in a variety of clusters including those infecting *M. smegmatis, Mycobacterium chelonae*, *Mycobacterium tuberculosis, Propionibacterium Freudenreichii*, *Gordonia terrae*, *Arthrobacter* sp., and *Rhodococcus erythropolis* (Fig. 6A; Table 2). We also identified candidates in Cluster X, encoding a different phamily of integrases with ~375 members (Fig. 6; Table 2).

**TABLE 2:** Phages using tRNA-dependent lysogeny

Cluster^*a*^	Example^*b*^	Host^*c*^	attB^*d*^	Core (bp)^*e*^	tRNA recon?^*f*^	Phage tRNA^*g*^	tRNA-dependent lysogeny^*h*^
AY	Hestia	*Arthrobacter* sp.	tRNA-Thr-CGT	52	Yes	No	No
BV	B22	*P. freudenreichii*	tRNA-Gly-CCC	42/44	Yes	No	No
BW	E1	*P. freudenreichii*	tRNA-Gly-CCC	42/44	Yes	No	No
CQ2	BrutonGaster	*G. terrae*	tRNA-Lys-TTT	11	No	Yes	Yes
CQ2	OneUp	*G. terrae*	tRNA-Ile2-CAT	10	No	Yes	Yes
CY2	BritBrat	*G. terrae*	tRNA-Gly-CCC	47	Yes	No	No
DL	Mollymur	*G. terrae*	tRNA-Ile2-CAT	9	No	Yes	Yes
DN1	Whitney	*G. terrae*	tRNA-Gly-TCC	51	Yes	No	No
DN2	Spooky	*G. terrae*	tRNA-Gly-CCC	47	Yes	No	No
DN4	Kuwabara	*G. terrae*	tRNA-Gly-TCC	47	Yes	No	No
F2	Yoshi	*M. smegmatis*	tRNA-Ile2-CAT (attB-13)	43/45	Yes	No	No
F2	Zapner	*M. smegmatis*	Not tRNA	ND	ND	No	No
FB	Kumotta	*Arthrobacter* sp.	tRNA-Met-CAT	51	Yes	No	No
K3	ShedlockHolmes	*M. smegmatis*	tRNA-Lys-CTT (attB-4)	48	Yes	No	No
K3	TribleTrouble	*M.smegmatis*	tRNA-Lys-TTT (attB-4)	43	Yes	No	No
K4	Fionnbharth	*M. smegmatis*	tRNA-Lys-TTT (attB-4)	10	No	Yes	Yes
K6	Cain	*M. smegmatis*	tRNA-Lys-TTT (attB-4)	11	No	Yes	Yes
K6	Hammy	*M. smegmatis*	tRNA-Lys-CTT (attB-2)	49	Yes	No	No
K6	Ximenita	*M. smegmatis*	tRNA-Lys-TTT (attB-4)	45	Yes	No	No
K7	Aminay	*M. smegmatis*	tRNA-Lys-CTT (attB-2)	33/36	Yes	No	No
K8	Boilgate	*M. smegmatis*	tRNA-Lys-TTT (attB-4)	38/43	Yes	No	No
L1	Lebron	*M. smegmatis*	tRNA-Lys-TTT (attB-4)	17	No	Yes	Yes
L2	Gabriela	*M. smegmatis*	tRNA-Lys-TTT (attB-4)	13	No	Yes	Yes
L3	Whirlwind	*M. smegmatis*	tRNA-Lys-TTT (attB-4)	9	No	Yes	Yes
L4	Chaser	*M. smegmatis*	tRNA-Lys-TTT (attB-4)	17	No	Yes	Yes
L5	Baudelaire	*M. chelonae*	tRNA-Lys-TTT (attB-4)	14	No	Yes	Yes
Sin	DS6A	*M. tuberculosis*	tRNA-Lys-CTT (attB-2)	49	Yes	No	No
Sin	Jace	*R. erythropolis*	tRNA-Gly-CCC	47	Yes	No	No
T	RonRayGun	*M. smegmatis*	tRNA-Lys-CTT (attB-2)	45/47	Yes	No	No
X	Gaia	*M. smegmatis*	tRNA-Leu-CAA (attB-11)	8	No	Yes	Yes
J	Omega	*M. smegmatis*	tRNA-Leu-CAA (attB-11)	44	Yes	No	Yes

^*a*^ Phage cluster/subcluster designation. All of the phage-encoded integrases are in the same phamily and are related to each other, except for Gaia (Cluster X) and Omega (Cluster J) that are in a different phamily.

^*b*^ Representative cluster/subcluster phages; note that not all members within a cluster encode closely related integrases.

^*c*^ Host species the phage was isolated on.

^*d*^ tRNA isotype and anticodon-overlapping *attB* site. *Mycobacterium attB* designations are shown in parentheses.

^*e*^ Number of base pairs shared between *attP* and *attB*. ND, not determined.

^*f*^ Yes, No, or ND, whether a functional tRNA is predicted to be present after integration at *attL* or *attR*.

^*g*^ Yes or No, whether a cognate tRNA to that overlapping *attB* is found in the phage genome.

^*h*^ Whether the phages use tRNA-dependent lysogeny.

Phages encoding Fionnbharth-related integrases are diverse (Fig. 6A), all use *attB* sites overlapping tRNA genes, and we identified additional examples of tRNA-dependent lysogeny (Table 2). However, not all use attB-4, and we identified examples using attB-11 and attB-13 (*G. terrae* equivalent) overlapping tRNA-Lys-CTT and tRNA-Ile2-CAT genes, respectively (Table 2). This suggests that tRNA-dependent lysogeny evolved more than once, and the phylogenetic relationship of the integrases is consistent with this (Fig. 6A). Cluster X (e.g., Gaia) and phages with this different phamily of integrases also integrate at *attB* sites overlapping tRNA genes, but the only instance of tRNA-dependent lysogeny we identified is phages Gaia and Nebkiss in Cluster X, which integrate at attB-11 overlapping a tRNA-Leu-CAA gene (Fig. 6; Table 2). Among the non-*Mycobacterium* phages using tRNA-dependent lysogeny, we identified candidates using *attB* sites overlapping tRNA-Lys-TTT and tRNA-Ile2-CAT (Table 2).

We note that closely related phages (i.e., within the same cluster or subcluster) vary in their integration systems. The *Gordonia* phages BrutonGaster and OneUp (both in Subcluster CQ2) use *attB* sites overlapping tRNA-Lys-TTT and tRNA-Ile2-CAT, respectively, neither reconstitutes the tRNA upon integration, and each encodes a tRNA cognate of the interrupted host tRNA (Fig. 6B and C). Similarly, *Gordonia* phages Mollymur and Daredevil (both in Cluster DL) integrate at tRNA-Ile2-CAT and tRNA-Lys-TTT genes, respectively, and neither reconstitute the tRNA upon integration; however, only Mollymur encodes its own tRNA (Fig. 6B and C), while Daredevil forms clear plaques as predicted (Fig. 6D). The 22 phages in Subcluster K6 are highly varied, and six (e.g., Cain) use tRNA-dependent lysogeny systems (Table 2; Fig. 6B and C), 12 (e.g., Ximenita) reconstitute the host tRNA when integrated (Table 2; Fig. 6C), and two (Hammy and DarthP) integrate at *attB* [attB-2; (14)] overlapping tRNA-Lys-CTT but do not use tRNA-dependent lysogeny; two K6 phages (Marshawn and TClif) use an integrase related to Subcluster K1 phages and use the tmRNA overlapping attB-9 (14, 30, 42). Omega and Gaia (Cluster J and X, respectively) code for related integrases, both are predicted to use attB-11 overlapping tRNA-Leu-CAA, but Omega reconstructs the tRNA gene (43) whereas Gaia does not, and it encodes its own tRNA-Leu-CAA gene (Fig. 6E and F). Sequencing the attachment junctions resulting from Gaia integration confirms integration at a short 8-bp common core (Fig. 6F).

## DISCUSSION

tRNA-dependent lysogeny provides new insights into the regulation of phage life cycles and is a notable departure from the well-studied phage lambda prototype. Lambda does not code for any tRNA genes (1), does not use an *attB* site overlapping a host tRNA gene, and its *E. coli* host has considerable tRNA redundancy. In this regard, it is atypical of the thousands of temperate phages and prophages of the Actinobacteria. Nonetheless, if lambda (or another prototype used for studying lysogeny) did use tRNA-dependent lysogeny, then studies to identify lysogeny requirements would have revealed clear plaque mutations in a tRNA gene, in addition to cI, cII, and cIII. This illustrates the diversity of regulatory systems used by temperate phages and the benefit of understanding this diversity more broadly.

In temperate phages using lambda-like systems for life cycle control, superinfection immunity and prophage integration are coupled but separable systems. Repressor and integrase both require cII to activate their expression, but integration is not required for the establishment of superinfection immunity (1). As such, integrase-defective phage mutants form turbid plaques, but when surviving cells are propagated and purified, they are not lysogenic. In tRNA-dependent lysogeny, superinfection immunity and prophage integration are linked in an unusual way, and although integrase-defective mutants form turbid plaques with superinfection immunity, phage tRNA-defective mutants form clear plaques, specifically because integration occurs, but the progeny are non-viable; integration thus becomes a lethal event. We note that previously described integration-dependent immunity systems (3) also differ from canonical lambda-like regulation, in that establishment of superinfection immunity is directly dependent on prophage integration, which facilitates re-structuring of the repressor gene needed for expression of the active form of the repressor (3). It is plausible that additional variations in how temperate life cycles are regulated remain to be discovered.

The variety of phages, their hosts, and the tRNA loci they use as *attB* attachment sites suggest that tRNA-dependent lysogeny is not uncommon among temperate phages of Actinobacteria and likely evolved independently more than once. However, the evolutionary relationships of tRNA-dependent lysogeny to canonical systems in which a host tRNA is reconstructed at an attachment junction following integration are not clear. Although tRNA-dependent lysogeny could have preceded canonical systems and simplified *attB* choice—as extended homology to retain tRNA functionality at *attL*/*attR* is not required—it is also plausible that loss of tRNA functionality from a lysogenic strain provided selection for acquisition of a tRNA gene with the same isotype in the phage genome. Conversely, acquisition of a tRNA of the same isotype as that of overlapping *attB* could have resulted in loss of selective pressure to maintain an *attP* core of sufficient length to reconstitute a functional tRNA. In addition, it has been suggested that phage-encoded tRNAs may counter bacterial defense systems that function by targeted degradation of host tRNAs (29), and *E. coli* PrrC specifically targets degradation of tRNA-Lys-TTT (44). It is thus plausible that selection of a tRNA gene required for lysogeny counteracts abortive infection mechanisms that degrade specific tRNA isotypes.

These tRNA-dependent lysogeny systems illuminate several unusual aspects of phage integration systems. A variety of phage-encoded tyrosine integrases have been characterized and typically cleave the DNA sites during recombination at scissile bonds spaced 6–8 bp apart with mismatches between *attP* and *attB* strongly deleterious to recombination (45, 46). It is unclear if the Fionnbharth Int and related integrases act similarly, but it is striking that among the secondary *attB* sites characterized, strong conservation is only seen with the three nucleotide positions (5′-CTT) at the left end of the common core. It is plausible that these integrases cleave their attachment site substrates with a three-base overlap region, or they may differentially tolerate base mismatches within a larger overlap region. We note that these integrases are lambda like in their overall organization and have an N-terminal “arm-type” DNA-binding domain, and pairs of putative arm-type DNA sites flank the *attP* common core. The general mechanisms of integration and excision are not expected to differ from the lambda prototype (2).

It is plausible that tRNA-dependent lysogeny is deployed by phages more broadly outside of the Actinobacteriophages. It was previously reported that integration of the Lj965 prophage resident in *Lactobacillus johnsonii* fails to reconstruct a tRNA at an attachment junction, and the prophage genome codes for a cognate tRNA that could complement its loss of function (47–49). Further investigation is needed to determine how prevalent the phenomenon of tRNA-dependent lysogeny is outside of the Actinobacteriophages.

## MATERIALS AND METHODS

### Bacterial strains and media

*M. smegmatis* strains were grown in Middlebrook 7H9 (Difco) supplemented with albumin dextrose complex and Tween80 (0.05%). Cultures were grown with shaking at 37°C 250 rpm. For growth on solid media, *M. smegmatis* cultures were grown on Middlebrook 7H10 media containing 0.2% dextrose, supplemented with 1 mM calcium chloride for phage infection (50). Middlebrook Top agar overlays used 7H9 Middlebrook and 0.35% Bacto Agar on 7H10 plates supplemented with 1 mM calcium chloride.

### Plasmid constructions

Plasmids used in this study are listed in Table S2. Plasmid pCG38 was constructed by PCR amplification of the Fionnbharth *int-attP* region to include the putative integrase binding sites (Fig. S3). PCR used 1 µL of phage lysate at 10^10^ pfu/mL and Q5 2xMaster Mix (NEB) under standard cycle conditions; primers are listed in Table S3. The PCR product (15–20 ng) was then used for a second round of PCR using primers to add homology flanking the Nde I site of vector pMOS-hyg. Nde I-linearized pMOS-hyg and PCR product were Gibson assembled, and transformants recovered in *E. coli* NEB 5α chemically competent cells (NEB). Plasmid pCG44 was constructed by PCR amplification of the Fionnbharth tRNA and its upstream region, addition of homology in a second round of PCR, and Gibson assembly with Hind III-linearized pCG38. The same strategy was used to construct the integration vectors described here, using phage-specific primers (Table S3) and *attP* organizations (Fig. S3 and S4). Cluster L phage tRNA genes were synthesized as gblocks (IDT), and these were inserted together with the putative promoter region of the Fionnbharth tRNA. Plasmid CG110 was similarly constructed by inserting a gblock with the predicted Fionnbharth tRNA promoter region into the linearized plasmid pLO73, such that the putative promoter is upstream of a synthetic RBS and the mcherry gene (34). Relative fluorescence units of mcherry expression from bacterial cultures grown for ~48 h and quantified in a 96-well plate using a plate reader.

### Preparation of competent cells and plasmid transformation into *M. smegmatis*

Competent cells were prepared by growing *M. smegmatis* to mid-log phase (OD ~0.7) pelleting cells at 5,000 × *g* at 4°C and washing with ice cold 10% glycerol three times. Cells were flash frozen on dry ice and stored at −80°C until use. For experiments comparing integration plasmids, three independent cultures were used to prepare competent cells. Competent cells were thawed on ice, and 100 ng of plasmid electroporated as described previously (51).

### Lysogen construction

Lysogens were isolated by plating bacterial cultures on solid media seeded with phage (~10^9^ PFU) incubated at 37°C, and surviving colonies were purified, grown in liquid culture, and tested for superinfection immunity and phage release as described previously (33). Lysogenization frequencies were determined relative to survivors on solid media without phage.

### Extraction of bacterial DNA

Cells from 1 mL bacterial cultures were collected by centrifugation, washed three times with 7H9 + Tween80 (to remove free phage from lysogenic cultures), resuspended in 0.6 mL nuclei lysis solution (Promega), and transferred to tubes containing Lysis matrix B beads (MP Biologicals). Cells were milled at 4,300 rpm for 1 min in a Benchmark Scientific BeadBug6 three times, cooling on ice for 2 min between times. The cell lysate was treated with RNAseA before adding 0.6 mL phenol-chloroform-isoamyl alcohol 24:23:1 (Thermo). Tubes were inverted to mix and centrifuged to separate aqueous and organic phases; the aqueous phases were collected and re-extracted similarly. DNA was precipitated from the final aqueous phase with isopropyl alcohol and 3 M sodium acetate; the precipitated DNA was collected by centrifugation, washed with 75% ethanol, air dried, and resuspended in 50–100 μL of 5 mM Tris-HCl pH 8.0. Illlumina sequencing was used to determine the integration locus of plasmid pCG46 in *M. smegmatis*.

### Isolation of phage DNA

Approximately 10^9^ to 10^10^ pfu in 0.5 mL of phage lysate was treated with lysis buffer (0.2% SDS, 0.05 mg/mL Proteinase-K, 12.5 mM EDTA) and incubated at 55°C for 15 min; 0.5 mL phenol:chloroform:isoamyl alcohol 24:23:1 (Thermo) was added, mixed, and centrifuged to separate aqueous and organic phases. The aqueous phase was collected and re-extracted similarly. Genomic DNA was precipitated from the aqueous phase by the addition of isopropyl alcohol and 3M sodium acetate, the DNA collected by centrifugation, washed with 75% ethanol, air dried, and resuspended in 50–100 μL of 5 mM Tris-HCl pH 8.0. Illlumina sequencing was used to confirm genotypes for Fionnbharth, Fionnbharth Δ5′tRNA, Fionnbharth Δ*int*, Fionnbharth Δ5′tRNA Δ*int*, Fionnbharth Δ*rep*, and Rumpelstiltskin.

### PCR

Primers are listed in Table S3. For PCR using bacterial colonies, a small part of a colony was picked into 20 µL 5 mM Tris-HCl pH 8, heated at 98°C for 10 min, and cooled. Two microliters of cell lysate were used as template for PCR using QuickLoad Taq 2xMaster Mix (NEB) with 20 µL total reaction volume. For other PCR reactions, ~30 ng DNA was used as template with *att*-specific primers (Table S3). PCR products were analyzed by agarose gel electrophoresis or by Sanger sequencing (Genewiz, Azenta Life Sciences).

### Phage-genome engineering

Phage DNA (100 ng) was co-electroporated with a synthetic DNA substrate (200 ng) into electrocompetent-recombineering cells as described previously (35). Primary plaques were screened by PCR to identify mixed plaques which were purified and retested by PCR; plaques were purified a third time and confirmed by PCR. BRED substrates (amplified from gblock DNA, Table S3) for Δ*int* and Δ*rep* mutants are 500 bp long with the deletion centrally located, with FionnbharthΔInt and FionnbharthΔ*rep* deleting coordinates 33,695–34,932 and 35,833–36,186, respectively. FionnbharthΔ5′tRNA mutant was constructed using a 200-bp substrate-deleting coordinates 30,911–30,919. MAMA-PCR (3) with NEB Quickload Taq polymerase was used to selectively amplify the mutant allele.

### Bioinformatics

Sequence alignments were made in CLC genomics workbench 23.0 (Qiagen). Phylogenetic trees were constructed with UPGMA and Kimura protein substitution with bootstrap = 100 replicates; amino acid sequences were trimmed using TrimAl (52); trees were visualized using FigTree v1.4.4 (http://tree.bio.ed.ac.uk/software/figtree/). Predictions of tRNA genes and structures were made using tRNAScan-SE v2.0 (53), and tRNA secondary structures visualized using RiboSketch (54). Phage-genome maps were made and visualized using Phamerator (16).

## Data Availability

Information on all phages used here including their accession numbers is available at phagesdb.org.

## SUPPLEMENTAL MATERIAL

Supplemental Materials mbio.03260-23-s0001.pdf Supplemental tables and figures.

ASM does not own the copyrights to Supplemental Material that may be linked to, or accessed through, an article. The authors have granted ASM a non-exclusive, world-wide license to publish the Supplemental Material files. Please contact the corresponding author directly for reuse.
