# Supplementary material for: Bacteriophage tRNA-dependent lysogeny: requirement of phage-encoded tRNA genes for establishment of lysogeny
Source: mBio. 2024 Jan 18;15(2):e03260-23. doi: 10.1128/mbio.03260-23 (PMC10865867; doi:10.1128/mbio.03260-23)
Supplement: Supplemental Materials — Supplemental tables and figures. [file mbio.03260-23-s0001.pdf]

Supplemental Materials

Table S1. Cluster L tRNA genes.

Table S2. Plasmids used in this study

Table S3. Synthetic DNAs used in this study.

Figure S1. *attP* sites of Fionnbharth and Boilgate. The sequence of the intergenic regions upstream of the integrases of Fionnbharth and Boilgate are shown. Predicted arm-type Integrase-binding sites are shown as boxes numbered P1-P4 for Fionnbharth and P1-P5 for Boilgate. Each phage has a pair of directly oriented arm-type sites both t the left and to the right of the common core; Boilate has an additional site (P3) to the right of the core in the indirect orientation. The *attP* cores are shown in bold-type. The N-terminal sequences of the integrase proteins are shown for Fionnbharth (Red) and Boilgate (yellow).

Figure S2. Constructions of Fionnbharth∆Int, Fionnbharth∆Rep, and a Fionnbharth∆Int∆5'tRNA double mutant. A. Construction of the Fionnbharth∆Int mutant. A 250 bp substrate for BRED mutagenesis with 125 bp of upstream and downstream of *int* (gene *45*) was co-electrporated with Fionnbharth genomic DNA and primary plaques recovered. Fourteen plaques were screened by PCR, as shown below, for the presence of the wild type 1.6 kbp product or the mutant allele product of ~500 bp. A single mixed primary plaque (#2) was identified, which was re-plated and 46 secondary plaques were picked and retested by PCR as shown below. Two homogenous phage mutants (+) were identified. Control (C) lanes include wild type phage DNA (-) and the BRED substrate (+). M, 1Kb-plus DNA ladder (NEB). **B**. Construction of the Fionnbharth∆Rep mutant. A 250 bp substrate for BRED mutagenesis with 125 bp of upstream and downstream of the repressor gene (*rep*, gene *45*) was co-electrporated

with Fionnbharth genomic DNA and primary plaques recovered. Four primary plaques were PCR amplified as shown below, all four of which amplified both wild type (0.8 kbp) and mutant (0.5 kbp) alleles. A primary mixed plaque was replated and twenty secondary plaques were PCR screened. A single plaque (#8) is homogenously mutant. Control (C) lanes include wild type phage DNA (-) and the BRED substrate (+). M, 1Kb plus ladder (NEB). **C**. Construction of a Fionnbharth Δ45 Δ5'tRNA double mutant. The same DNA substrate used to construct the Fionnbharth Δ5'tRNA mutant (Fig. 3A) was co-electrporated with FionnbharthΔint genomic DNA and seven primary plaques were screened by PCR as shown to the right. These primers cannot readily distinguish the wild type and mutant alleles, but MAMA-PCR selectively amplified the mutant allele, and showed that two primary plaques include the desired mutant. Controls include wild type Fionnbharth DNA and FionnbharthΔ5'tRNA DNA (+ and – respectively). A mutant plaque was picked and purified, and secondary plaques screen by PCR as shown below. Two secondary mutant plaques were identified by MAMA-PCR (bottom right panel). Both were picked and purified.

Figure S3. Immunity of secondary site lysogens of FionnbharthΔ5'tRNA. Ten-fold serial dilutions of Fionnbharth (F'bharth*, a Δ45Δ47 lytic mutant) and control phage Whirlwind (Cluster L3) were spotted onto lawns of *M. smegamtis* mc^2155, a FionnbharthΔInt lysogen (only one of seven tested is shown; all behaved identically) and seven FionnbharthΔ5'tRNA lysogens after colony purification. The FionnbharthΔInt lysogens are not maintained through colony purification as the phage cannot integrate into the chromosome and superinfection immunity to Fionnbharth is lost. All seven FionnbharthΔ5'tRNA lysogens retain superinfection immunity to Fionnbharth.

Figure S4. *attP* sites of LeBron, Whirlwind, and Baudelaire. The sequence of the intergenic region upstream of the integrases of Cluster L phages LeBron (L1), Whirlwind (L3) and

Baudelaire (L5) are shown. Predicted arm-type Integrase-binding sites are shown as boxes numbered P1-P5 for LeBron, P2 and P3 for Whirlwind, and P1-P3 for Baudelaire. The *attP* cores are shown in bold-type. The N terminal amino acid sequences of the integrase proteins are shown for LeBron (magenta) and Whirlwind (Cyan) and Baudelaire (Yellow). LeBron has only a single arm-type site to the left of the core (P1), and three directly oriented sites (P3, P4 and P5) to the right of the core. Whirlwind has a pair of directly oriented sites to the right of the common core (P2 and P3), but we have not identified arm-type sites to the left of the core. Baudelaire has a single arm-type site (P1) to the left of the core and a pair of directly oriented sites (P2, P3) to the right of the core.

Figure S5. tRNA genes in Cluster L phages. Genome maps of representative Cluster L phages LeBron (L1); Gabriela (L2), Rumpelstiltskin (L2); Whirlwind (L3); Bromden and Chaser (L4) and Baudelaire (L5) are shown. The genome maps show the extreme right ends of the phage genomes with the tRNA genes shown as small grey boxes; each tRNA and its anticodon are indicated in black type, except for tRNA-Lys-TTT genes which are shown in red bold type. Nucleotide similarity between pairs of genomes is spectrum colored with violet being the most similar and red being the least similar above a threshold BLASTN E value of 10^{-4}. Colored boxes indicate predicted protein-coding genes colored according to their phamily assignment. Genes and tRNAs above and below the genome ruler are transcribed rightwards and leftwards, respectively.

Table S1. tRNAs identified in Cluster L genomes using tRNAScan-SE 2.0

	Subcluster	tRNA pos[1]	tRNA Start[2]	tRNA End[3]	tRNA type[4]	Anti Codon[5]
LeBron	L1	1	60863	60936	Leu	CAA
LeBron	L1	2	60938	61011	Thr	CGT
LeBron	L1	3	61299	61371	Lys	CTT
LeBron	L1	4	61383	61465	Tyr	GTA
LeBron	L1	5	61476	61548	Trp	CCA
LeBron	L1	6	61550	61623	Leu	TAG
LeBron	L1	7	61625	61695	His	GTG
LeBron	L1	8	62874	62947	Cys	GCA
LeBron	L1	9	63302	63230	Lys	TTT
Gabriela	L2	1	61992	62064	Thr	CGT
Gabriela	L2	2	62293	62366	Pro	TGG
Gabriela	L2	3	62488	62560	Trp	CCA
Gabriela	L2	4	62672	62753	Tyr	GTA
Gabriela	L2	5	63168	63241	Leu	TAG
Gabriela	L2	6	63237	63316	His	GTG
Gabriela	L2	7	63317	63390	Gln	CTG
Gabriela	L2	8	63507	63580	Gly	TCC
Gabriela	L2	9	63625	63697	Lys	CTT
Gabriela	L2	10	64884	64954	Cys	GCA
Gabriela	L2	11	65228	65297	Asn	GTT
Gabriela	L2	12	65566	65494	Lys	TTT
Rumpelstilskin	L2	1	62640	62709	Lys	CTT
Whirlwind	L3	1	39380	39462	Tyr	GTA
Whirlwind	L3	2	63038	63109	Thr	CGT
Whirlwind	L3	3	63469	63542	Pro	TGG
Whirlwind	L3	4	63884	63957	Gly	TCC
Whirlwind	L3	5	64013	64085	Trp	CCA
Whirlwind	L3	6	64087	64160	Leu	TAG
Whirlwind	L3	7	64162	64235	His	GTG
Whirlwind	L3	8	64513	64587	Gln	CTG
Whirlwind	L3	9	64717	64789	Lys	CTT
Whirlwind	L3	10	65920	65991	Asn	GTT
Whirlwind	L3	11	66887	66816	Lys	CTT
Whirlwind	L3	12	66070	65998	Lys	TTT
Bromden	L4	1	64661	64743	Leu	CAA
Bromden	L4	2	64745	64816	Thr	CGT
Bromden	L4	3	65316	65389	Pro	TGG
Bromden	L4	4	65682	65754	Trp	CCA
Bromden	L4	5	65756	65837	Tyr	GTA
Bromden	L4	6	66159	66232	Leu	TAG
Bromden	L4	7	66234	66304	His	GTG
Bromden	L4	8	66306	66378	Ile	GAT
Bromden	L4	9	66443	66514	Gln	CTG
Bromden	L4	10	66516	66589	Gly	TCC
Bromden	L4	11	66639	66711	Lys	CTT
Bromden	L4	12	67014	67085	Ala	TGC
Bromden	L4	13	68770	68840	Asn	GTT
Chaser	L4	1	63020	63102	Leu	CAA
Chaser	L4	2	63112	63183	Thr	CGT
Chaser	L4	3	63814	63890	Pro	TGG
Chaser	L4	4	64183	64259	Trp	CCA
Chaser	L4	5	64962	65035	Leu	TAG
Chaser	L4	6	65037	65109	His	GTG
Chaser	L4	7	65111	65185	Gln	CTG
Chaser	L4	8	65300	65373	Gly	TCC
Chaser	L4	9	65423	65495	Lys	CTT
Chaser	L4	10	66888	66958	Cys	GCA
Chaser	L4	11	67665	67735	Asn	GTT
Chaser	L4	12	68004	67932	Lys	TTT

Baudelaire	L5	1	64811	64894	Leu	CAA
Baudelaire	L5	2	65132	65205	Thr	CGT
Baudelaire	L5	3	65256	65331	fMet	CAT
Baudelaire	L5	4	65391	65464	Ile	GAT
Baudelaire	L5	5	65670	65743	Pro	TGG
Baudelaire	L5	6	65822	65905	Tyr	GTA
Baudelaire	L5	7	65960	66032	Phe	GAA
Baudelaire	L5	8	66038	66111	Trp	CCA
Baudelaire	L5	9	66188	66262	His	GTG
Baudelaire	L5	10	66264	66338	Gln	CTG
Baudelaire	L5	11	66350	66422	Glu	CTC
Baudelaire	L5	12	66810	66882	Gly	TCC
Baudelaire	L5	13	66896	66972	Lys	CTT
Baudelaire	L5	14	66998	67070	Lys	TTT
Baudelaire	L5	15	67088	67162	Ala	TGC
Baudelaire	L5	16	67362	67432	Cys	GCA
Baudelaire	L5	17	67562	67633	Asn	GTT

Table S1 is a modified output from analysis of a subset of the Cluster L genomes using tRNAScan-SE 2.0 (Chan PP, Lin BY, Mak AJ, Lowe TM. 2021. tRNAscan-SE 2.0: improved detection and functional classification of transfer RNA genes. Nucleic acids research 49:9077-9096)

[1]Number represents the numerical order at which the tRNA appears in the genome, with 1 meaning the tRNA is first.
[2]Start coordinate for the tRNA in the phage genome.
[3]End coordinate for the tRNA in the phage genome.
[4]Most likely type of tRNA identified based on sequence conservation and secondary structure.
[5]Anticodon tRNA of isotype

Table S2. Plasmids used in this study

Plasmid[1]	Backbone[2]	Resistance[3]	features/notes[4]	Reference[5]
pMOS-Hyg	pMos-Blue	HygR	OriE, MCS	(1)
pCG38	pMOS-hyg	HygR	Fionnbharth attP+ Int	this study
pCG44	pCG38	HygR	Fionnbharth tRNA-Lys-TTT + promoter	this study
pCG69	pMOS-hyg	HygR	Gabriela attP + Int	this study
pCG70	pMOS-hyg	HygR	Chaser attP + Int	this study
pCG71	pMOS-hyg	HygR	Baudelaire attP +Int	this study
pCG72	pMOS-hyg	HygR	Boilgate attP + Int	this study
pCG74	pCG69	HygR	Gabriela tRNA-Lys-TTT with Fionnbharth tRNA promoter	this study
pCG75	pCG70	HygR	Chaser tRNA-Lys-TTT with Fionnbharth tRNA promoter	this study
pCG76	pCG71	HygR	Baudelaire tRNA-Lys-TTT with Fionnbharth tRNA promoter	this study
pJV53	pLAM12	KanR	plasmid used for BRED engineering	(2, 3)
pLO73	pTTP1B	KanR	Calibrated promoter set no promoter	(4)
pLO74	pTTP1B	KanR	Calibrated promoter set hsp60 promoter	(4)
pLO75	pTTP1B	KanR	Calibrated promoter set BPs P_R	(4)
pLO76	pTTP1B	KanR	Calibrated promoter set BPs P_R	(4)
pLO77	pTTP1B	KanR	Calibrated promoter set BPs P_R	(4)
pLO78	pTTP1B	KanR	Calibrated promoter set BPs P_R	(4)
pLO93	pTTP1B	KanR	Calibrated promoter set BPs P_R	(4)
pCG110	pLO73	KanR	Fionnbharth tRNA promoter	this study

[1]Name of plasmid following standard convention.

[2]Parent plasmid that was used to generate the construct.

[3] Antibiotic resistance cassette HygR indicates Hygromycin resistance KanR indicates Kanamycin resistance.

[4] Additional features present in the backbone or parent plasmids or notes for use of plasmid.

[5] References for plasmid.

1. Singh S, Rockenbach K, Dedrick RM, VanDemark AP, Hatfull GF. 2014. Cross-talk between Diverse Serine Integrases. Journal of Molecular Biology 426:318-331.
2. van Kessel JC, Marinelli LJ, Hatfull GF. 2008. Recombineering mycobacteria and their phages. Nat Rev Microbiol 6:851-7.
3. Marinelli LJ, Piuri M, Swigonová Z, Balachandran A, Oldfield LM, van Kessel JC, Hatfull GF. 2008. BRED: a simple and powerful tool for constructing mutant and recombinant bacteriophage genomes. PLoS One 3:e3957.
4. Oldfield LM, Hatfull GF. 2014. Mutational analysis of the mycobacteriophage BPs promoter PR reveals context-dependent sequences for mycobacterial gene expression. Journal of bacteriology 196:3589-3597.

Table S3. Synthetic DNAs using in this study

Primer sequence 5' to 3'	Primer name	Purpose
CGCTGGCTCCAATCCTTTG	Fionnbharth attP int amplify F	amplify Fionnbharth integrase and attP and attL
CTAACTCAGCAGCCGAGTG	Fionnbharth attP int amplify R	amplify Fionnbharth integrase and attP and attR
GTAATACGACTCACTATAGGGCNNNNNN	T7 primer with random hexamer	Paired with Fionnbharth attP int amplify R to find attR
GCGCGCAGCGGCTTTCTG	Boilgate Int_attP_F	amplify Boilgate integrase and attP and attL
TCACGCCCGACGATCAGG	Boilgate_attP_R	amplify Boilgate integrase and attP and attR
TCTCTCCGGGGCGCGTTCG	attB-4 F	amplify smeg attB-4 and integrating phages attL
CGTGACGTGACGGTCACCACGAACA	attB-4 R	amplify smeg attB-4 and integrating phages attR
TCTAGAGGATCTACTAGTCATATGCGCTGGCTCCAATCCTTTG	pMos homology Fionnbharth attP int F	add homology to Fionnbharth attP Int for pMOS-hyg
ACTTTAGATTGATTTATCCATATGCTAACTCAGCAGCCGAGTG	pMos homology Fionnbharth attP int R	add homology to Fionnbharth attP Int for pMOS-hyg
CCGGGTTGACCCGATCCACCGT	Fionn_tRNA amplify F	amplify Fionnbharth tRNA
GTCAACCCACACGATACACGAGCGTG	Fionn_tRNA amplify R	amplify Fionnbharth tRNA
ATACGACTCACTATAGGGAATGGTGCCCCGCCGGAGTT	add homology HindIII to tRNA_lysF	add homology to Fionnbharth tRNA for pMOS-hyg
AGTCGACCTGCAGGCATGCAGGTTTGTGGCTCCTAACTTCTCACGTACC	add homology HindIII to tRNA_lysR	add homology to Fionnbharth tRNA for pMOS-hyg
TCTAGAGGATCTACTAGTCATATGGCGCGCAGCGGCTTTCTG	Boilgate Int_attP_pmos-hom_F	add homology to Boilgate attP Int for pMOS-hyg
ACTTTAGATTGATTTATCCATATGTCACGCCCGACGATCAGG	Boilgate_attP_pmos-hom_R	add homology to Boilgate attP Int for pMOS-hyg
ATGAGACCGGCTGATTGTGCATGGC	Fionn_tRNA amplify Forward check (anneals outside)	Primer for Fionnbharth tRNA WT or deletion F
GAGCGGCGTCGCTATAGTGTGTCTCAAT	Junction primer del 5' tRNA	Primer for Fionnbharth del 5' tRNA MAMA PCR
TGGCCGCGAGATCCCCTCG	Fionnbharth 2º attB1 F	amplify and sequence 2º attB1 and 2º attL1
AGCAGTTGCCGCTGGTGCAC	Fionnbharth 2º attB1 R	amplify and sequence 2º attB1 and 2º attR1
GACGCGCGACACTAGCTGCAG	Fionnbharth 2º attB2 F	amplify and sequence 2º attB2 and 2º attR2
CGGCCATCAGCCCGAGCAC	Fionnbharth 2º attB2 R	amplify and sequence 2º attB2 and 2º attL2
CCCGGGGATACAGCCCTGACC	Fionnbharth 2º attB3 F	amplify and sequence 2º attB3 and 2º attL3
GGCATGCAGACCGCGATGACG	Fionnbharth 2º attB3 R	amplify and sequence 2º attB3 and 2º attR3
TCCTCGAGCCGCTCCGGC	Fionnbharth 2º attB4 F	amplify and sequence 2º attB4 and 2º attL4
CCAGGCCGACCGCCTGCT	Fionnbharth 2º attB4 R	amplify and sequence 2º attB4 and 2º attR4
GCGCGCGTGTCTCCTACC	Gabriela Int_attP_F	amplify Gabriela and attP and attL
CTAACCGGACGGGAGCAG	Gabriela Int_attP_R	amplify Gabriela integrase and attP and attR
GCGCGTGTCTCCTACCGT	Chaser Int_attP_F	amplify Chaser integrase and attP and attL
CTAAACGTTAGGGAGCAGAGCG	Chaser Int_attP_R	amplify Chaser integrase and attP and attR
GTCAGTTGCCTTTCGGGG	Baudelaire Int_attP_F	amplify Baudelaire integrase and attP and attL
CTAAACGCGGTCCAATACC	Baudelaire_attP_R	amplify Baudelaire integrase and attP and attR
TCTAGAGGATCTACTAGTCATATGGCGCGCGTGTCTCCTACC	Gabriela Int_attP_pmos-hom_F	add homology to Gabriela attP Int for pMOS-hyg
ACTTTAGATTGATTTATCCATATGCTAACCGGACGGGAGCAG	Gabriela Int_attP_pmos-hom_R	add homology to Gabriela attP Int for pMOS-hyg
TCTAGAGGATCTACTAGTCATATGGCGCGTGTCTCCTAC	Chaser Int_attP_pmos-hom_F	add homology to Chaser attP Int for pMOS-hyg

ACTTTAGATTGATTTATCCATATGCTAAACGTTAGGGAGCAGAG	Chaser Int_attP_pmos-hom_R	add homology to Chaser attP Int for pMOS-hyg
TCTAGAGGATCTACTAGTCATATGGTCAGTTGCCTTTCGGGG	Baudelaire Int_attP_pmos-hom_F	add homology to Baudelaire attP Int for pMOS-hyg
ACTTTAGATTGATTTATCCATATGCTAAACGCGGTCCAATACC	Baudelaire_attP_pmos-hom_R	add homology to Baudelaire attP Int for pMOS-hyg
GGCTGAAAGCGCGGTGAGGGACTTC	Fionnbharth del int F check	Fionnbharth integrase deletion check primer
CTGGCTCCAATCCTTTGCTCGTTG	Fionnbharth del int F	Fionnbharth integrase gblock amplification primer
CCGCAAGCGCCAATCATCTGGAC	Fionnbharth del int R	Fionnbharth integrase gblock amplification primer
TGCCGCAAGCGCCAATCATCTG	Fionnbharth del rep check R	Fionnbharth repressor deletion check primer
ACCAGCGTGAGTGATTGCCCGCGTTCCCAG	Fionnbharth del rep F	Fionnbharth repressor gblock amplification primer
TGCGGCGCAGCAGTTTTGCGATTG	Fionnbharth del rep R	Fionnbharth repressor gblock amplification primer
GCCGGACGGCGGGAGAAA	pLO73 linearize F	PCR linearization of pLO73 for placement of promoter
TAGATTTAAAGATCTGGTACCGCGGCC	pLO73 linearize R	PCR linearization of pLO73 for placement of promoter

gBLOCKS

gblock sequence 5' to 3'	gblock name	Purpose
CTGGCTCCAATCCTTTGCTCGTTGGGTGCAGGCCGGCACTGTACCCGAGATTGAGGCGTTTTTGGGTACATGTTTGGGTACACCGCTACGAGCACGGTGCTTCTGACCTGCGGTTTTACGGTGGATTGCACCGGAATCGGCTGATTAAAAGTTCGTCGCCTCGGCGTGTCGACCAGGCATAACACCAGGTCACGCTACCCCGTAGTGCTGTGTAGTGCTTGGTAGGAGTACCGGCTTGGGTACACCTGTGCGGCTGCTGAGTTAGCAGTCGGTGGCCGGTCGTCGCCCGCGTTCGCGTCGGCGCGGCTGCTTCGGCTGGTGGTGGATGGCGTTCCACAGCTTCGTGAACGAGCGGGTTTTCTGCTGCCAGCTCCGCACCGCCGAGATGACGAGCAGCACGGCGCTGATGCCGGTGAGCGTCCAGATGATTGGCGCTTGCGG	Fionnbharth del Int	gblock integrase deletion
ACCAGCGTGAGTGATTGCCCGCGTTCCCAGTAGCTGCCCCAACCTAAGCGCCGTACCCACAGCGCCAGTAGGGTGAATGTGACAACCGTGGACACGAACGTAGACAGCATCATCGGCGTAGTTCCCTAACCATCTAAGGAATTGAGCCGCAACCTTTTTCGGGTTGTCGACCGTATGGGGGGCTATGTGTGCCGCTGCTACACAACATAGTCCAGATTTATGGACAAAGCAACTCGATTGACGTGCAGATTCTCCAGATCATCGGGCAACACGCTGTCAAGATATCCGTAATGATGTCCACAAACCTGGACACAGTGTTGTACCTTCGTAGGCATGCCAGCCCCTCAGACCTACGAACTCCGATGGAATCCGGAAGCAATCGCAAAACTGCTGCGCCGCA	Fionnbharth del rep	gblock repressor deletion
CCGGGTTGACCCGATCCACCGTCTAGGAAAGTTGATGGTGCCCCGCCGGAGTTTCGATCTCCGTACCCGCCGGTTAAAAGCCGGCTGCTCTTCCGATTGAGACACACTATAGCGACG	Fionnbharth del tRNA	gblock 5'tRNA-Lys-TTT deletion

CCGCTCTCCGGTGCCTAATCAGCGCCGGGGGGCGGCGTTTCTGTGTTGAGTGGTCTACACGCTCGTGTATCGTGTGGGTTGAC		
ATACGACTCACTATAGGGAAAGCTTGGGTGCCCCCACTAGGGATCGAACCTAGGACCCGAAGATTAAAAGTCTCCTGCTCTACCAACTGAGCTATAGGGGCGACACACTATAGCGACGCCGCTCTCCGGTGCCTAATCAGCGCCGGGGGGCGGCGTTTCTGTGTTGAGTGGTCTACACGCTCGTGTATCGTGTGGGTTGACCTACACGGGTACGTGAGAAGTTAGGAGCCACAAACCAAGCTTGCATGCCTGCAGGTCGACT	Gabriela tRNA with Fionnbharth promoter pMOS homology	gblock Gabriela tRNA under Fionnbharth tRNA promoter and flanking homology to pMOS-hyg HindIII restriction site
ATACGACTCACTATAGGGAAAGCTTGGTGCCCCCACTAGGGATCGAACCTAGGACCCGATGATTAAAAGTCACCTGCTCTGCCAACTGAGCTATAGGGGCGACACACTATAGCGACGCCGCTCTCCGGTGCCTAATCAGCGCCGGGGGGCGGCGTTTCTGTGTTGAGTGGTCTACACGCTCGTGTATCGTGTGGGTTGACCTACACGGGTACGTGAGAAGTTAGGAGCCACAAACCAAGCTTGCATGCCTGCAGGTCGACT	Chaser tRNA with Fionnbharth promoter pMOS homology	gblock Chaser tRNA under Fionnbharth tRNA promoter and flanking homology to pMOS-hyg HindIII restriction site
ATACGACTCACTATAGGGAAAGCTTGTACGCCGTGTAGGGCTCGAACCTACGACCTACTGATTAAAAGTCAGCAGCTCTACCAACTGAGCTAACGGCGCGACACACTATAGCGACGCCGCTCTCCGGTGCCTAATCAGCGCCGGGGGGCGGCGTTTCTGTGTTGAGTGGTCTACACGCTCGTGTATCGTGTGGGTTGACCTACACGGGTACGTGAGAAGTTAGGAGCCACAAACCAAGCTTGCATGCCTGCAGGTCGACT	Baudelaire tRNA with Fionnbharth promoter pMOS homology	gblock Baudelaire tRNA under Fionnbharth tRNA promoter and flanking homology to pMOS-hyg HindIII restriction site
GTACCAGATCTTTAAATCTAGGTTTGTGGCTCCTAACTTCTCACGTACCCGTGTAGGTCAACCCACACGATACACGAGCGTGTAGACCACTCAACACAGAAACGCCGCCCCCCGGCGCTGATTAGGCACCGGAGAGCGGCGTCGCTATAGTGTGTCGCCGGACGGCGGGAGAAAGG	Fionnbharth tRNA promoter with Homology to pLO73	gblock Fionnbharth tRNA promoter sequence with flanking homology to pLO73 for unsertion upstream of the TM4 capsid RBS

Fionnbharth

```
                P1             P2
33501 GCGTTTTTGGGTACATGTTTGGGTACACCGCTACGAGCACGGTGCTTCTGACCTGCGGTTTTACGGTGGATTGCACCGGAATCGGCTGATTAAAAGTTCG
      ....:....|....:....|....:....|....:....|....:....|....:....|....:....|....:....|....:....|....:....|
      CGCAAAAACCCATGTACAAACCCATGTGGCGATGCTCGTGCCACGAAGACTGGACGCCAAAATGCCACCTAACGTGGCCTTAGCCGACTAATTTTCAAGC

                                                                                          P3         P4
33601 TCGCCTCGGCGTGTCGACCAGGCATAACACCAGGTCACGCTACCCCGTAGTGCTGTGTAGTGCTTGGTAGGAGTACCGGCTTGGGTACACCTGTGGGTAC
      ....:....|....:....|....:....|....:....|....:....|....:....|....:....|....:....|....:....|....:....|
      AGCGGAGCCGCACAGCTGGTCCGTATTGTGGTCCAGTGCGATGGGGCATCACGACACATCACGAACCATCCTCATGGCCGAACCCATGTGGACACCCATG

        int    M  A  T  K  R  R  A  P  G  E  G  G  L  F  K  R  A  D  G  M  W  V  G  R  V  D  V  P  T  A
33701 AGTCGCGGGCATGGCAACGAAGCGACGCGCGCCCGGCGAGGGCGGTCTGTTCAAGCGCGCCGATGGCATGTGGGTCGGACGTGTAGACGTTCCTACAGCC
      ....:....|....:....|....:....|....:....|....:....|....:....|....:....|....:....|....:....|....:....|
      TCAGCGCCCGTACCGTTGCTTCGCTGCGCGCGGGCCGCTCCCGCCAGACAAGTTCGCGCGGCTACCGTACACCCAGCCTGCACATCTGCAAGGATGTCGG
```

Boilgate

```
                                                                                                   P1
33101 GCGGCTTTCTGAAAGCTGAGGTTTGGTGCGGGTCCGCCCGCCGAACCCCCTCAAGATCGGCGGGCGGACCCGCGTGGATGCAACTGTACCTCAGTTTGGG
      ....:....|....:....|....:....|....:....|....:....|....:....|....:....|....:....|....:....|....:....|
      CGCCGAAAGACTTTCGACTCCAAACCACGCCCAGGCGGGCGGCTTGGGGGAGTTCTAGCCGCCCGCCTGGGCGCACCTACGTTGACATGGAGTCAAACCC

                  P2
33201 TACACCTTTGGGTACACCTCCGAATCTTCATGATCACCGCCACCCTTTCTGACCTGCGGGTTTTCGGTGCCCCTACTAGGACTCGAACCTAGGACCTCCG
      ....:....|....:....|....:....|....:....|....:....|....:....|....:....|....:....|....:....|....:....|
      ATGTGGAAACCCATGTGGAGGCTTAGAAGTACTAGTGGCGGTGGGAAAGACTGGACGCCCAAAAGCCACGGGGATGATCCTGAGCTTGGATCCTGGAGGC

                                                                                           P3        P4
33301 GATTAAAAGATCGACGAAGTAGGGTAGCCTACCAGGCGTTTTAGCAGGTCGGCGGCTTCCGTAGCACTACTTAGGACTGCACTGTACCCAACGCTTGGGT
      ....:....|....:....|....:....|....:....|....:....|....:....|....:....|....:....|....:....|....:....|
      CTAATTTTCTAGCTGCTTCATCCCATCGGATGGTCCGCAAAATCGTCCAGCCGCCGAAGGCATCGTGATGAATCCTGACGTGACATGGGTTGCGAACCCA

                P5          int M  P  K  P  R  R  N  A  R  R  A  P  G  E  G  S  L  F  K  R  S  D  G  M  W  V
33401 ACAGTTTTGGGTACAGTAGACGAATGCCGAAACCGCGTCGCAACGCCCGCCGCGCCCCCGGAGAGGGGAGCCTGTTCAAACGCTCCGACGGCATGTGGGT
      ....:....|....:....|....:....|....:....|....:....|....:....|....:....|....:....|....:....|....:....|
      TGTCAAAACCCATGTCATCTGCTTACGGCTTTGGCGCAGCGTTGCGGGCGGCGCGGGGGCCTCTCCCCTCGGACAAGTTTGCGAGGCTGCCGTACACCCA
```

Figure S1

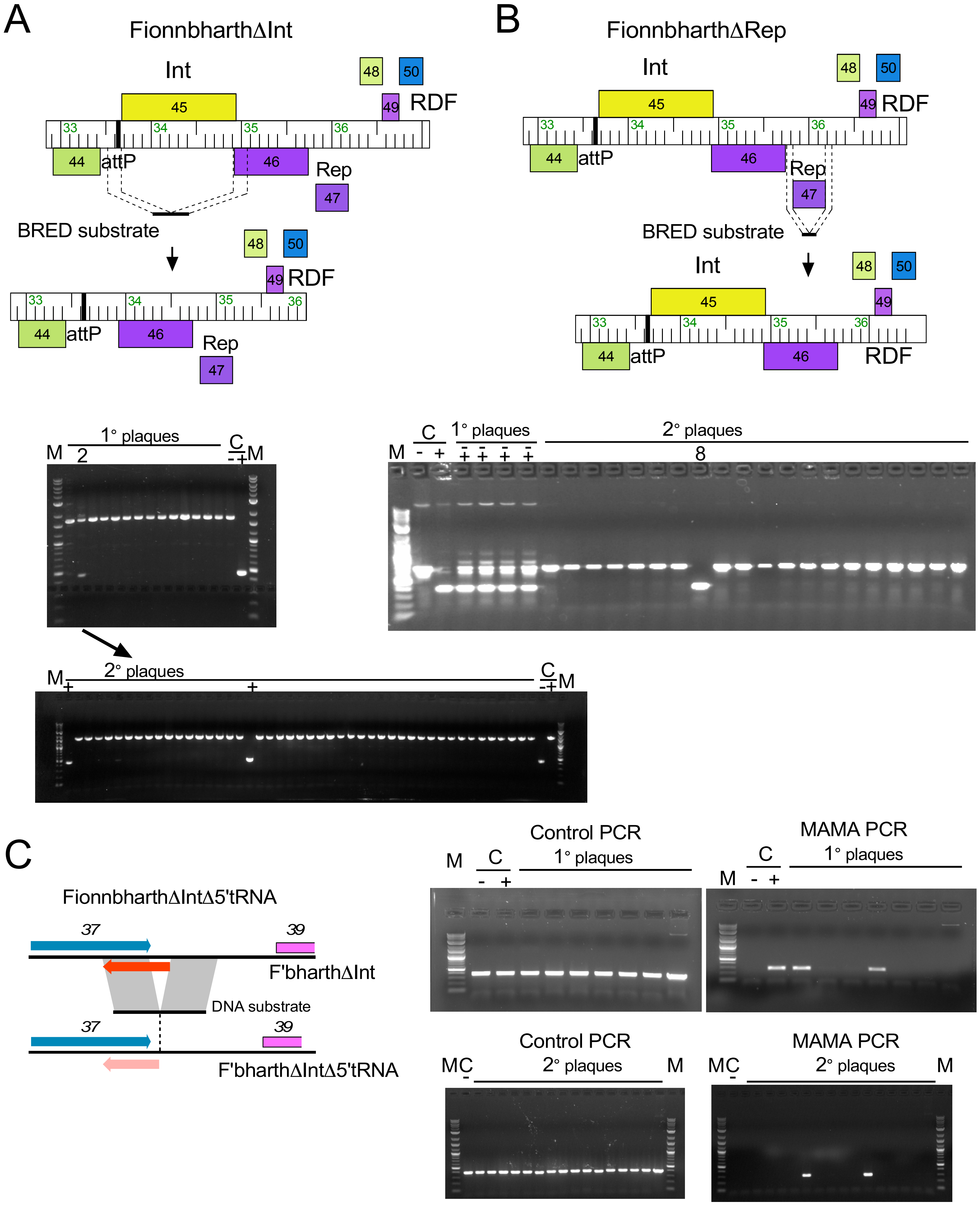
A
FionnbharthΔInt
Int
45
48
50
49
RDF
33
34
35
36
44
attP
46
Rep
47
BRED substrate
1° plaques
M
2
C
- +
M
2° plaques
B
FionnbharthΔRep
1° plaques
2° plaques
8
C
Control PCR
MAMA PCR
FionnbharthΔIntΔ5'tRNA
37
39
F'bharthΔInt
DNA substrate
F'bharthΔIntΔ5'tRNA

Figure S2

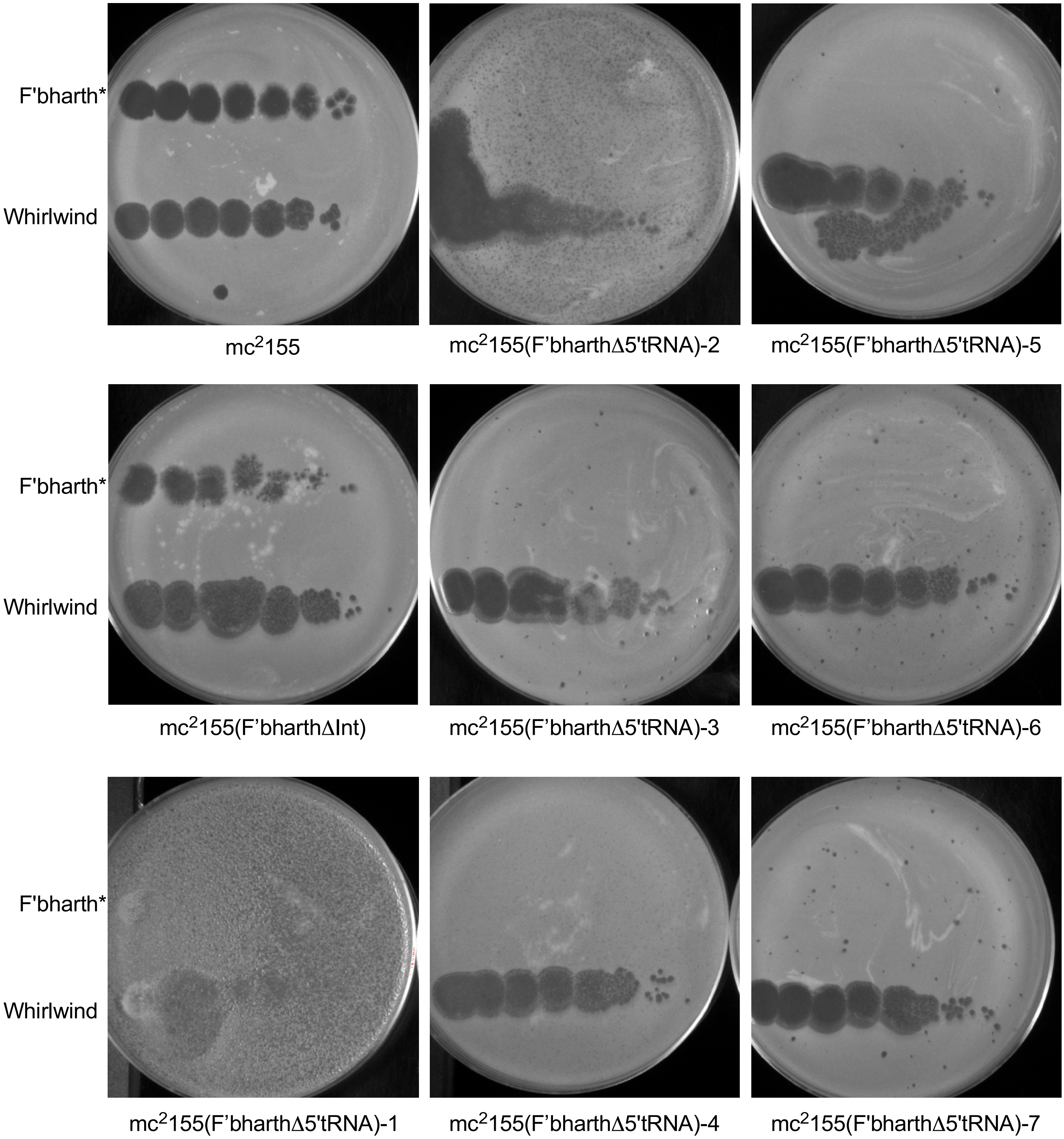

F'bharth*
Whirlwind
mc2155
mc2155(F'bharthΔ5'tRNA)-2
mc2155(F'bharthΔ5'tRNA)-5
mc2155(F'bharthΔInt)
mc2155(F'bharthΔ5'tRNA)-3
mc2155(F'bharthΔ5'tRNA)-6
mc2155(F'bharthΔ5'tRNA)-1
mc2155(F'bharthΔ5'tRNA)-4
mc2155(F'bharthΔ5'tRNA)-7

Figure S3

LeBron

```
                                                                                                    P1
30001 TCATAGTTCTCTTACTGTTTGAGGGTGTCTGAAACATGTCCGCAGGTGAGGCCCTTAACCCGTTCTGACCAGCCACGATCTGATTCTTAGTTGACACCTG
      ....:....|....:....|....:....|....:....|....:....|....:....|....:....|....:....|....:....|....:....|
      AGTATCAAGAGAATGACAAACTCCCACAGACTTTGTACAGGCGTCCACTCCGGGAATTGGGCAAGACTGGTCGGTGCTAGACTAAGAATCAACTGTGGAC

30101 TGTTGACACTTGACAGAGAAAACCGCTCTGACCAGGCATTTCTTAGAAGAGTCTGAGGATTAAAAGTCCGTAGAGGCACCCCCTGCACATGCTGGTGTAG
      ....:....|....:....|....:....|....:....|....:....|....:....|....:....|....:....|....:....|....:....|
      ACAACTGTGAACTGTCTCTTTTGGCGAGACTGGTCCGTAAAGAATCTTCTCAGACTCCTAATTTTCAGGCATCTCCGTGGGGGACGTGTACGACCACATC
                                                                               P3          P4          P5
30201 TCTGTCAAACATCCAGCTAACGAGGGTTTAAGTCTCGTATGGCACGCTGTGGCACTGCTGGATAGTTGACACCTTAGTTGACACCTAGTTGACACCTGGG
      ....:....|....:....|....:....|....:....|....:....|....:....|....:....|....:....|....:....|....:....|
      AGACAGTTTGTAGGTCGATTGCTCCCAAATTCAGAGCATACCGTGCGACACCGTGACGACCTATCAACTGTGGAATCAACTGTGGATCAACTGTGGACCC

        int V  A  E  T  D  S  K  I  K  R  R  L  R  G  D  G  G  L  T  Y  D  K  S  R  G  L  Y  I  G  I  /
30301 AGGGTGCGGAGTGGCCGAGACGGACTCGAAAATTAAGAGACGCCTAAGGGGTGATGGAGGTCTCACCTACGACAAATCGCGGGGTCTCTACATCGGCATC
      ....:....|....:....|....:....|....:....|....:....|....:....|....:....|....:....|....:....|....:....|
      TCCCACGCCTCACCGGCTCTGCCTGAGCTTTTAATTCTCTGCGGATTCCCCACTACCTCCAGAGTGGATGCTGTTTAGCGCCCCAGAGATGTAGCCGTAG
```

Whirlwind

```
31301 TTCTGAAATATTAGAAAACCCTAAGGGCCGGTTTGCGGTGCGTCGGCGCGTCCGCAGGTCCCGGAGCTATACCTATCCCTAGCTGCGGCGATGCCCCAGG
      ....:....|....:....|....:....|....:....|....:....|....:....|....:....|....:....|....:....|....:....|
      AAGACTTTATAATCTTTTGGGATTCCCGGCCAAACGCCACGCAGCCGCGCAGGCGTCCAGGGCCTCGATATGGATAGGGATCGACGCCGCTACGGGGTCC

31401 CGGGGGTCCGTAAGTGATAGTTTTAGGTATAGCGACCGCGAAAAACGCTCTGACCTGCATGTTCTTAGGGCCACCAGAGGCTTAAAAGTCCCCACATAGG
      ....:....|....:....|....:....|....:....|....:....|....:....|....:....|....:....|....:....|....:....|
      GCCCCCAGGCATTCACTATCAAAATCCATATCGCTGGCGCTTTTTGCGAGACTGGACGTACAAGAATCCCGGTGGTCTCCGAATTTTCAGGGGTGTATCC
                                                                              P2        P3
31501 ACCCCGCACAACCTGCTAGAATCTGTCAAATGCCCAGCTAAGCGCCTTAGCGTACGCCTATGCGTGCCTATGCAGGCACACGCTAAGGTATAGCGCTCAG
      ....:....|....:....|....:....|....:....|....:....|....:....|....:....|....:....|....:....|....:....|
      TGGGGCGTGTTGGACGATCTTAGACAGTTTACGGGTCGATTCGCGGAATCGCATGCGGATACGCACGGATACGTCCGTGTGCGATTCCATATCGCGAGTC

                                                int V  T  D  D  T  E  K  T  K  R  R  N  R  G  D  G  G  L  F
31601 GTATAGTTGGGCCGGTTCCGGCACCGAGTGGAGGGTCAGAAAAGTGACAGACGACACAGAGAAAACCAAGAGACGCAACAGGGGTGACGGTGGCCTGTTC
      ....:....|....:....|....:....|....:....|....:....|....:....|....:....|....:....|....:....|....:....|
      CATATCAACCCGGCCAAGGCCGTGGCTCACCTCCCAGTCTTTTCACTGTCTGCTGTGTCTCTTTTGGTTCTCTGCGTTGTCCCCACTGCCACCGGACAAG
```

Baudelaire

```
          P1
33901 GATAGATGATACTTCTGATGATACTGGCGACGCGCTTAGCGTCTGCGATGCCTCTACCTGCATGGTTACGTCGCGTGTAACCCCTGGCCCGTGGATTAAA
      ....:....|....:....|....:....|....:....|....:....|....:....|....:....|....:....|....:....|....:....|
      CTATCTACTATGAAGACTACTATGACCGCTGCGCGAATCGCAGACGCTACGGAGATGGACGTACCAATGCAGCGCACATTGGGGACCGGGCACCTAATTT

34001 AGTCCGTAGTGCATTTAGGTAGCTCACCTGCTAGAGTGCTGTCAACAGCGCAGGTCAATGGCTGTTCCGGAGTCCACGCTAGCCCACGCTAGTGCCGGGT
      ....:....|....:....|....:....|....:....|....:....|....:....|....:....|....:....|....:....|....:....|
      TCAGGCATCACGTAAATCCATCGAGTGGACGATCTCACGACAGTTGTCGCGTCCAGTTACCGACAAGGCCTCAGGTGCGATCGGGTGCGATCACGGCCCA

       P2          P3                                         int M  V  V  A  G  Q  K  N  K  R  R  E  K  G  D  G  G
34101 AGATGATACCGATAGATGATACTTACCAGCCACAGGGAGCTAGCTAACGATGGTCGTTGCCGGTCAGAAAAATAAGAGACGTGAGAAGGGTGACGGGGGC
      ....:....|....:....|....:....|....:....|....:....|....:....|....:....|....:....|....:....|....:....|
      TCTACTATGGCTATCTACTATGAATGGTCGGTGTCCCTCGATCGATTGCTACCAGCAACGGCCAGTCTTTTTATTCTCTGCACTCTTCCCACTGCCCCCG
```

Figure S4

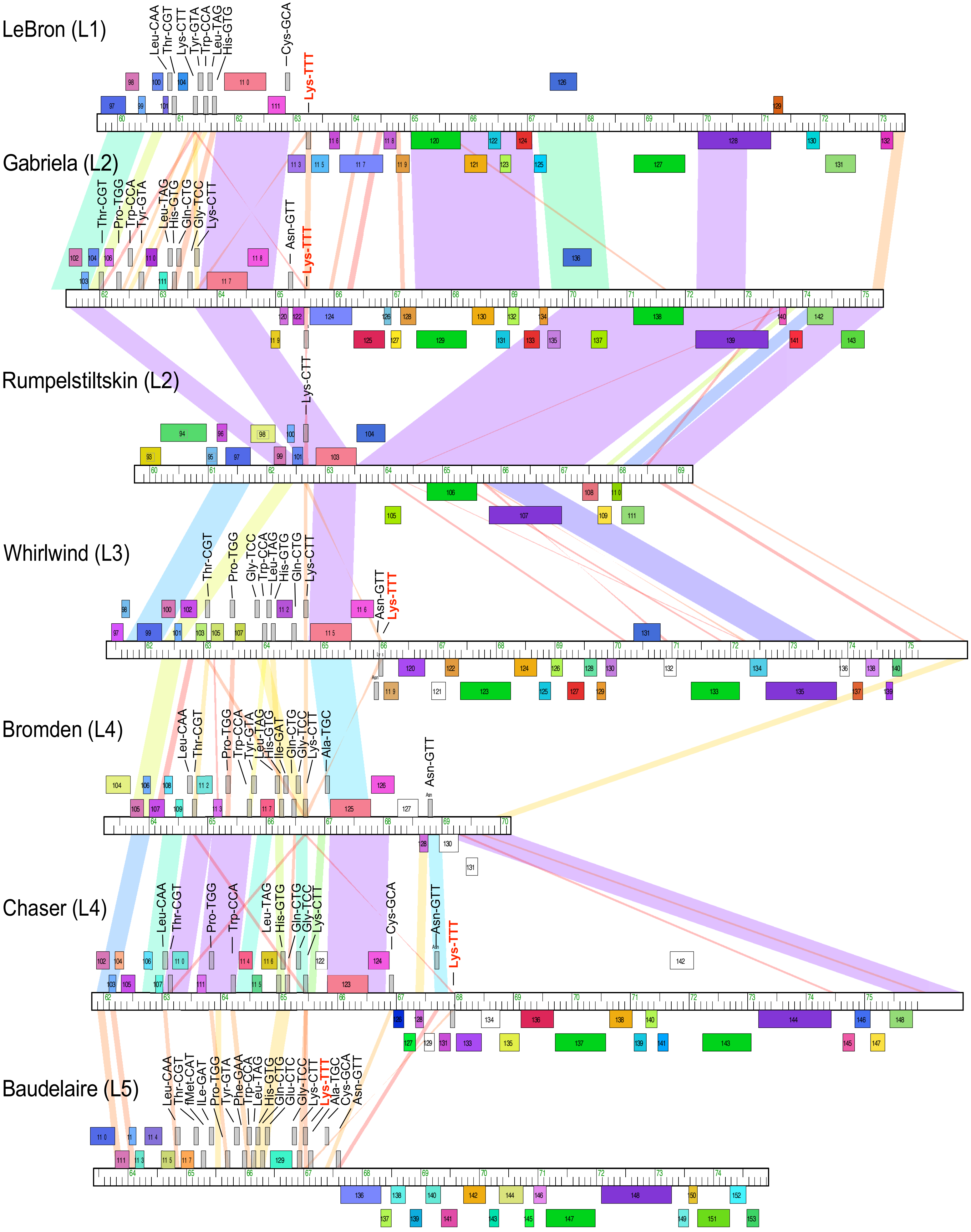

LeBron (L1)
Gabriela (L2)
Rumpelstiltskin (L2)
Whirlwind (L3)
Bromden (L4)
Chaser (L4)
Baudelaire (L5)

Figure S5
